# Baf155 regulates skeletal muscle metabolism via HIF-1a signaling

**DOI:** 10.1371/journal.pbio.3002192

**Published:** 2023-07-21

**Authors:** Jong-Seol Kang, Dongha Kim, Joonwoo Rhee, Ji-Yun Seo, Inkuk Park, Ji-Hoon Kim, Daewon Lee, WonUk Lee, Ye Lynne Kim, Kyusang Yoo, Sunghwan Bae, Jongkyeong Chung, Rho Hyun Seong, Young-Yun Kong

**Affiliations:** 1 School of Biological Sciences, Seoul National University, Seoul, South Korea; 2 Department of Anatomy, College of Medicine, The Catholic University of Korea, Seoul, South Korea; 3 Institute of Molecular Biology and Genetics, Seoul National University, Seoul, South Korea; King’s College London, UNITED KINGDOM

## Abstract

During exercise, skeletal muscle is exposed to a low oxygen condition, hypoxia. Under hypoxia, the transcription factor hypoxia-inducible factor-1α (HIF-1α) is stabilized and induces expressions of its target genes regulating glycolytic metabolism. Here, using a skeletal muscle-specific gene ablation mouse model, we show that Brg1/Brm-associated factor 155 (Baf155), a core subunit of the switch/sucrose non-fermentable (SWI/SNF) complex, is essential for HIF-1α signaling in skeletal muscle. Muscle-specific ablation of Baf155 increases oxidative metabolism by reducing HIF-1α function, which accompanies the decreased lactate production during exercise. Furthermore, the augmented oxidation leads to high intramuscular adenosine triphosphate (ATP) level and results in the enhancement of endurance exercise capacity. Mechanistically, our chromatin immunoprecipitation (ChIP) analysis reveals that Baf155 modulates DNA-binding activity of HIF-1α to the promoters of its target genes. In addition, for this regulatory function, Baf155 requires a phospho-signal transducer and activator of transcription 3 (pSTAT3), which forms a coactivator complex with HIF-1α, to activate HIF-1α signaling. Our findings reveal the crucial role of Baf155 in energy metabolism of skeletal muscle and the interaction between Baf155 and hypoxia signaling.

## Abstract 2

A number of subunits of the SWI/SNF chromatin remodeling complex have been shown to play a role in muscle development. Building on this work, in this study the authors investigate the contribution of a core SWI/SNF subunit, Baf155 in muscle function. The authors report that in mice, loss of Baf155 does not affect gross muscle development, but leads to increased oxidative metabolism and enhanced endurance capacity. These effects are mediated by Baf155 regulation of HIF-1a signaling.

## Introduction

Skeletal muscle is a high-energy–demanding organ and uses adenosine triphosphate (ATP) for contraction [1]. Due to the increase of metabolic rate by over 100-fold during exercise compared to the resting state [2], ATP needs to be supplied sufficiently to continue the exercise. Glucose metabolism is one of the ATP-generating pathways in skeletal muscle and consists of glycolysis and oxidation. Oxidation generates ATP slower but more efficiently than glycolysis [3,4] and supplies a major part of ATP for muscle contraction during prolonged endurance exercise [5,6]. In addition, the increased oxidation results in the enhancement of endurance exercise capacity [7]. These results imply that the balance between glycolysis and oxidation is crucial for exercise capacity. Accordingly, research on biological pathways regulating glucose metabolism in the skeletal muscle is essential for understanding the physiological mechanisms that contribute to the exercise function of skeletal muscles.

The heterodimeric transcription factor hypoxia-inducible factor (HIF), which is composed of an alpha subunit (HIF-1α, HIF-2α, or HIF-3α) and a beta subunit (HIF-1β, known as ARNT), is primarily activated under hypoxia [8]. Among the 3 alpha subunits, HIF-1α is known to be involved in regulating anaerobic glucose metabolism [9] and control glycolysis, which contributes to exercise capacity of the skeletal muscle [10–13]. Furthermore, muscle-specific loss of HIF-1α results in increased oxidation due to the reduced expressions of HIF-1α target genes and enhances endurance exercise capacity [7]. These results show the crucial role of HIF-1α signaling in energy metabolism and exercise capacity of skeletal muscle. However, notwithstanding the physiological importance of HIF-1α signaling in skeletal muscle, the molecular mechanisms regulating the activation of this signaling are not fully understood.

Switch/sucrose non-fermentable (SWI/SNF) complex is an ATP-dependent chromatin remodeler regulating the DNA binding of transcriptional complexes [14]. Mammalian SWI/SNF complex consists of catalytic ATPase subunit (Brahma; Brm or Brahma-related gene 1; Brg1) and other 9–12 subunits known as Brg1/Brm-associated factors (Bafs). ATPase subunits contribute to the function of SWI/SNF complex through their catalytic activity, and other Bafs have been considered structural proteins, only acting to stabilize the complex. However, recent studies revealed that some Bafs also serve key roles in transcriptional regulations by interacting with other transcription factors [15–17]. These fundamental roles of Bafs are also observed in the skeletal muscle [18,19]. Hence, research on the distinctive functions of each Baf subunit is important to understand the functional mechanisms of SWI/SNF complex. Baf155, one of the Baf subunits, is classified as a core subunit due to its general existence in all mammalian SWI/SNF complexes [20,21]. Although Baf155 is known as the structural protein protecting degradations of other subunits [22,23], the unique role of Baf155 in contributing to transcriptional regulations have not yet been studied.

In this study, we investigated the potential role of Baf155 in the skeletal muscle. Genetic ablation of Baf155 did not affect the formation and growth of the skeletal muscle. However, mice with Baf155 ablated skeletal muscle showed enhanced endurance exercise capacity. In addition, we revealed that this mouse model also showed increased oxidative metabolism and intramuscular ATP level. These results indicate that Baf155 is dispensable for the development and maturation but essential for the energy metabolism of skeletal muscle. Our chromatin immunoprecipitation (ChIP) analysis showed that Baf155 is involved in energy metabolism via HIF-1α signaling by mediating the DNA binding of HIF-1α. In addition, we also revealed that this regulatory function requires the DNA binding of pSTAT3, which is indispensable for HIF-1α signaling because it forms a transcriptional complex with HIF-1α. In summary, we identified the crucial role of Baf155 in skeletal muscle and revealed the functional mechanism of Baf155 in the energy metabolism of skeletal muscle.

## Results

### Baf155 ablation does not affect the stability of other components of the SWI/SNF complex in the skeletal muscle

**Fig 1:**
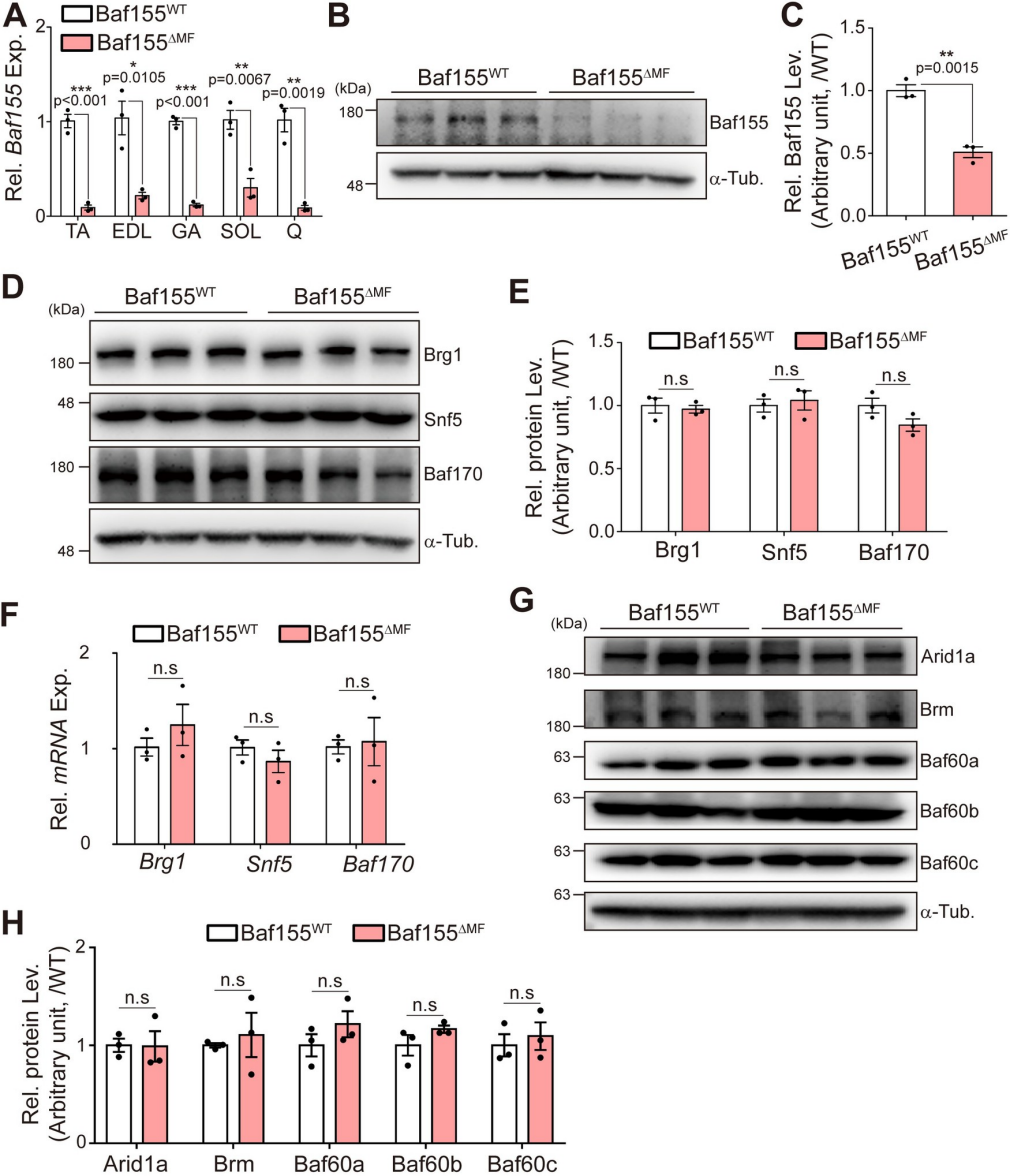
Baf155 ablation does not affect the stability of components of SWI/SNF complex in the skeletal muscle. **(A)** RT-qPCR analysis of the expression of *Baf155* in hind limb skeletal muscles (TA, EDL, GA, SOL, Q) of Baf155^WT^ and Baf155^ΔMF^ mice (*n* = 3 mice per genotype). **(B, C)** Representative immunoblotting analysis of Baf155 (B) and the densitometric quantification of relative Baf155 protein level in the Q muscle of Baf155^ΔMF^ mice compared to Baf155^WT^ mice (*n* = 3 mice per genotype) (C). **(D, E)** Representative immunoblotting analysis of Brg1, Snf5, and Baf170 (D) and the densitometric quantification of relative level of each indicated protein in Q muscle of Baf155^ΔMF^ mice compared to Baf155^WT^ mice (*n* = 3 mice per genotype) (E). **(F)** RT-qPCR analysis of the expressions of *Brg1*, *Snf5*, and *Baf170* in Q muscle of Baf155^WT^ and Baf155^ΔMF^ mice (*n* = 3 mice per genotype). **(G, H)** Representative immunoblotting analysis of Arid1a, Brm, Baf60a, Baf60b, and Baf60c (G) and the densitometric quantification of relative protein level of each indicated protein in Q muscle of Baf155^ΔMF^ mice compared to Baf155^WT^ mice (*n* = 3 mice per genotype) (H). Each lane in immunoblotting analysis (B, D, and G) indicates each mouse (biological replicate), and each dot in the graphs (A, C, E, F, and H) represents each mouse (biological replicate). Data are presented as mean ± SEM of biological replicates. Statistical analyses were performed using unpaired Student’s *t* test (n.s., not significant; *P < 0.05, **P < 0.01, ***P < 0.001 versus Baf155^WT^ control). The data underlying this figure can be found in S1 Data. Arid1a, AT-rich interaction domain 1a; Baf60, Brg1/Brm-associated factor 60; Baf155, Brg1/Brm-associated factor 155; Baf170, Brg1/Brm-associated factor 170; Brg1, Brahma-related gene 1; Brm, Brahma; EDL, extensor digitorum longus; GA, gastrocnemius; MF, myofiber; RT-qPCR, reverse transcription quantitative real-time PCR; SEM, standard error of the mean; Snf5, sucrose non-fermentable 5; SOL, soleus; TA, tibialis anterior; WT, wild type; Q, Quadriceps.

To investigate the function of Baf155 in the skeletal muscle, we specifically ablated *Baf155* by crossing *Baf155*^floxed/floxed^ mice with *MCK-*Cre (MCK: muscle creatine kinase) transgenic mice [24,25] (*MCK*-Cre; *Baf155*^f/f^, hereafter Baf155^ΔMF^). We confirmed the reduced levels of mRNA and protein in hind limb skeletal muscles using reverse transcription-quantitative PCR (RT-qPCR) and western blot, respectively (Fig 1A–1C). Since Baf155 stabilizes other subunits of the SWI/SNF complex [22,23], we investigated the protein levels of major components of this complex in Baf155^ΔMF^ skeletal muscle. Contrary to previous reports, despite the reduction of Baf155, other subunits in Baf155^ΔMF^ skeletal muscle remained comparable to the wild-type control (*Baf155*^*f/f*^, hereafter Baf155^WT^) (Fig 1D–1F). In addition, other components of the SWI/SNF complex were also comparable between Baf155^WT^ and Baf155 ^ΔMF^ mice (Fig 1G–1H). These results showed that Baf155 ablation did not affect the stability of other subunits in the skeletal muscle and implied that the SWI/SNF complex containing Baf155 was not formed in Baf155^ΔMF^ skeletal muscle.

### Baf155 is dispensable for the development and maturation of skeletal muscle

Since our result suggests the absence of SWI/SNF complex containing Baf155 in Baf155^ΔMF^ skeletal muscles, we first analyzed their gross morphologies to investigate whether the absence of this complex affects the development and maturation of skeletal muscles. We compared total body weight and the size and weight of skeletal muscles between Baf155^WT^ and Baf155^ΔMF^ mice but could not observe any differences (S1A–S1C Fig). We also performed a dual-energy X-ray absorptiometry (DEXA) scan to precisely analyze the percentage of body composition (lean, fat, and body fluid), but there was no difference between Baf155^WT^ and Baf155^ΔMF^ mice (S1D Fig). In addition, to analyze the histological characteristics, we performed hematoxylin and eosin (HE) and immunohistochemistry (IHC) analyses. From these experiments, we observed that the location of myonuclei and the number and size of myofibers were comparable between Baf155^WT^ and Baf155^ΔMF^ mice (S1E–S1H Fig). Considering that myofibers drive the transition of proliferating MuSCs to the quiescent state during postnatal maturation [26], we also quantified the number of MuSCs and observed similar numbers in both Baf155^WT^ and Baf155^ΔMF^ mice (S1E and S1H Fig). Based on these results, we concluded that Baf155 is dispensable for the development and maturation of the skeletal muscle.

### Baf155 ablation in skeletal muscle enhances endurance exercise capacity

**Fig 2:**
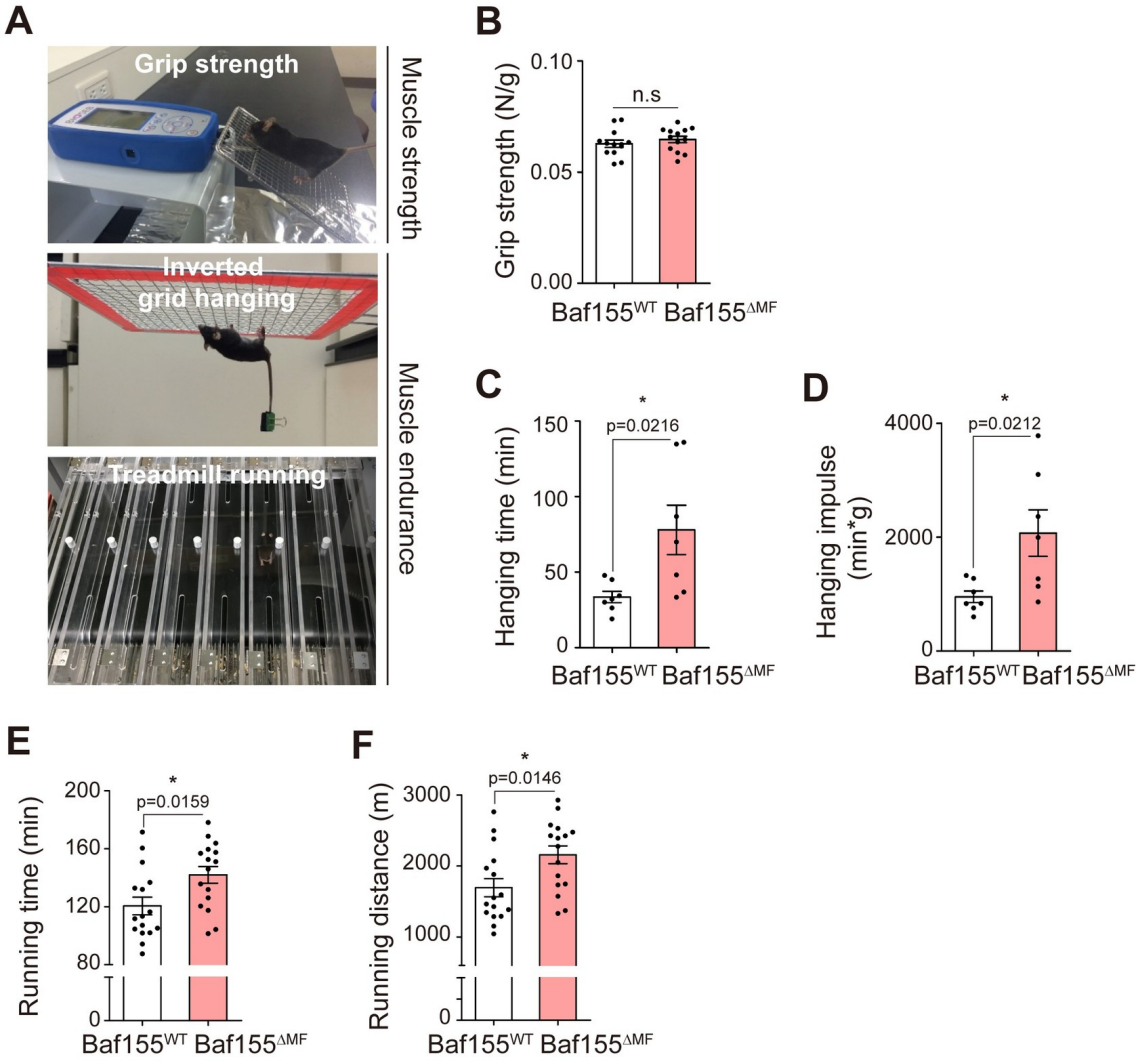
Baf155 ablation in skeletal muscle enhances endurance exercise capacity. **(A)** Representative images of exercise tests. The grip strength test measures muscle strength, and the inverted grid hanging and the treadmill running tests measure muscle endurance. **(B)** The measurement values of grip strength (N/g) of Baf155^WT^ and Baf155^ΔMF^ mice. The measured value (N) was normalized to body weight (g) (*n* = 13 mice per genotype). **(C, D)** The measurement values of inverted grid hanging test. Total hanging time (min) (C) and hanging impulse (min*g), which is the value of hanging time normalized to body weight to consider the effect of body weight (D) (*n* = 7 mice per genotype). **(E, F)** The measurement values of treadmill running test. Total running time (min) (E) and total running distance (m) (F) (*n* = 16 mice per genotype). Each dot in the graphs (B, C, D, E, and F) represents each mouse (biological replicate). Data are presented as mean ± SEM of biological replicates. Statistical analyses were performed using unpaired Student’s *t* test (n.s., not significant; *P < 0.05 versus Baf155^WT^ control). The data underlying this figure can be found in S1 Data. Baf155, Brg1/Brm-associated factor 155; m, meter; MF, myofiber; min, minutes; N, Newton; g, gram.

Next, we assessed exercise capacity, the physiological function of skeletal muscle. Considering that genetic changes result in the alteration of muscle function [27], Baf155 ablation could affect exercise capacity. To verify this possibility, we estimated 2 categories of exercise capacity, strength and endurance (Fig 2A). In a four-limb grip strength test, which measures muscle strength, Baf155^ΔMF^ mice showed comparable grip strength compared to Baf155^WT^ mice (Fig 2B). This result indicated that Baf155 ablation did not affect the strength generation of skeletal muscle. However, interestingly, Baf155^ΔMF^ mice showed enhanced exercise capacity in an inverted-grid hanging test, which measures endurance capacity [28,29]. Baf155^ΔMF^ mice endured hanging for a longer time than Baf155^WT^ mice (Fig 2C). In addition, to consider the effect of body weight, we calculated the hanging impulse (min*g; time multiplied by body weight) and observed a significant increment in Baf155^ΔMF^ mice (Fig 2D). These results indicated that Baf155^ΔMF^ mice had an enhancement in the endurance exercise capacity compared to Baf155^WT^ mice. To confirm the enhanced endurance capacity, we also performed a treadmill running test. As expected, Baf155^ΔMF^ mice showed significant increases in total running time and running distance to exhaustion compared to Baf155^WT^ mice, by more than 20 min and 25%, respectively (Fig 2E and 2F). Together, our exercise tests revealed that the ablation of Baf155 in skeletal muscle specifically enhanced endurance exercise capacity without affecting acute generation of strength.

Since *MCK*-Cre transgenic mice also show Cre activity in cardiac muscle [24,30–32], we validated the Baf155^ΔMF^ mouse model by investigating the expressions of Baf155 in various organs. As expected, Baf155 showed a significantly reduced mRNA level in limb muscle and cardiac muscle. However, its expression was comparable between Baf155^WT^ and Baf155^ΔMF^ mice in the other tested organs, such as liver, kidney, lung, and thymus (S2A Fig). In line with mRNA level, Baf155 protein level was also significantly reduced in limb muscle and cardiac muscle but not in the other organs of Baf155^ΔMF^ mice compared to Baf155^WT^ mice (S2B and S2C Fig). These results suggested the possible contribution of Baf155 ablation in cardiac muscle to the enhanced exercise capacity of Baf155^ΔMF^ mice. To exclude this possibility, we examined whether Baf155 ablation in cardiac muscle affects endurance exercise capacity. By crossing *Baf155*^f/f^ mice with *Myh6*-MerCreMer transgenic mice [33] (*Myh6*-MerCreMer; *Baf155*^f/f^, hereafter Baf155^ΔCMF^, Myh6: myosin heavy chain 6), we ablated Baf155 in cardiac muscle and verified the specific ablation in cardiac muscle, not in limb muscle, by RT-qPCR after tamoxifen treatment (S2D and S2E Fig). In addition, we also confirmed the ablation of Baf155 in the cardiac muscle of Baf155^ΔCMF^ mice by western blot (S2F and S2G Fig). Despite the ablation of Baf155 in cardiac muscle, Baf155^ΔCMF^ mice showed a similar weight of cardiac muscle compared to Baf155^WT^ mice (S2H Fig). In contrast with Baf155^ΔMF^ mice, Baf155^ΔCMF^ mice did not show the enhancement of endurance exercise in the treadmill running test (S2I and S2J Fig). These results indicated that not only was Baf155 dispensable for cardiac-muscle development, but also its ablation in cardiac muscle was irrelevant to the enhanced endurance exercise capacity of Baf155^ΔMF^ mice.

These intriguing observations prompted us to investigate skeletal muscle-intrinsic alterations in Baf155^ΔMF^ mice. We first analyzed myofiber type composition, which is one of the factors determining the exercise capacity [19,34,35], by performing the IHC analysis targeting myosin heavy chains (MyHC; MyHC1/2a and MyHC2b, which are expressed in slow oxidative/fast oxidative and fast glycolytic myofiber, respectively) [36]. We observed the comparable proportions of each fiber type between Baf155^WT^ and Baf155^ΔMF^ mice (S3A and S3B Fig) and confirmed these results by RT-qPCR (S3C Fig). Furthermore, since the increased mitochondrial oxidation could enhance the endurance exercise capacity without fiber type transition [37,38], we next examined the oxidation capacity of mitochondria by analyzing nicotinamide adenine dinucleotide hydrogen (NADH) [39,40]. However, we could not observe a difference in the number of NADH-positive fibers and the intensity of NADH staining (S3D–S3F Fig). We further investigated expressions of genes related to the mitochondrial function by RT-qPCR and observed similar levels in hind limb skeletal muscle from Baf155^WT^ and Baf155^ΔMF^ mice (S3G and S3H Fig). These results implied that the enhanced exercise capacity of Baf155^ΔMF^ mice was not due to changes in fiber type composition or mitochondrial function.

### JAK/STAT signaling is inhibited due to the reduced DNA binding of STAT3 in Baf155^△MF^ skeletal muscle

**Fig 3:**
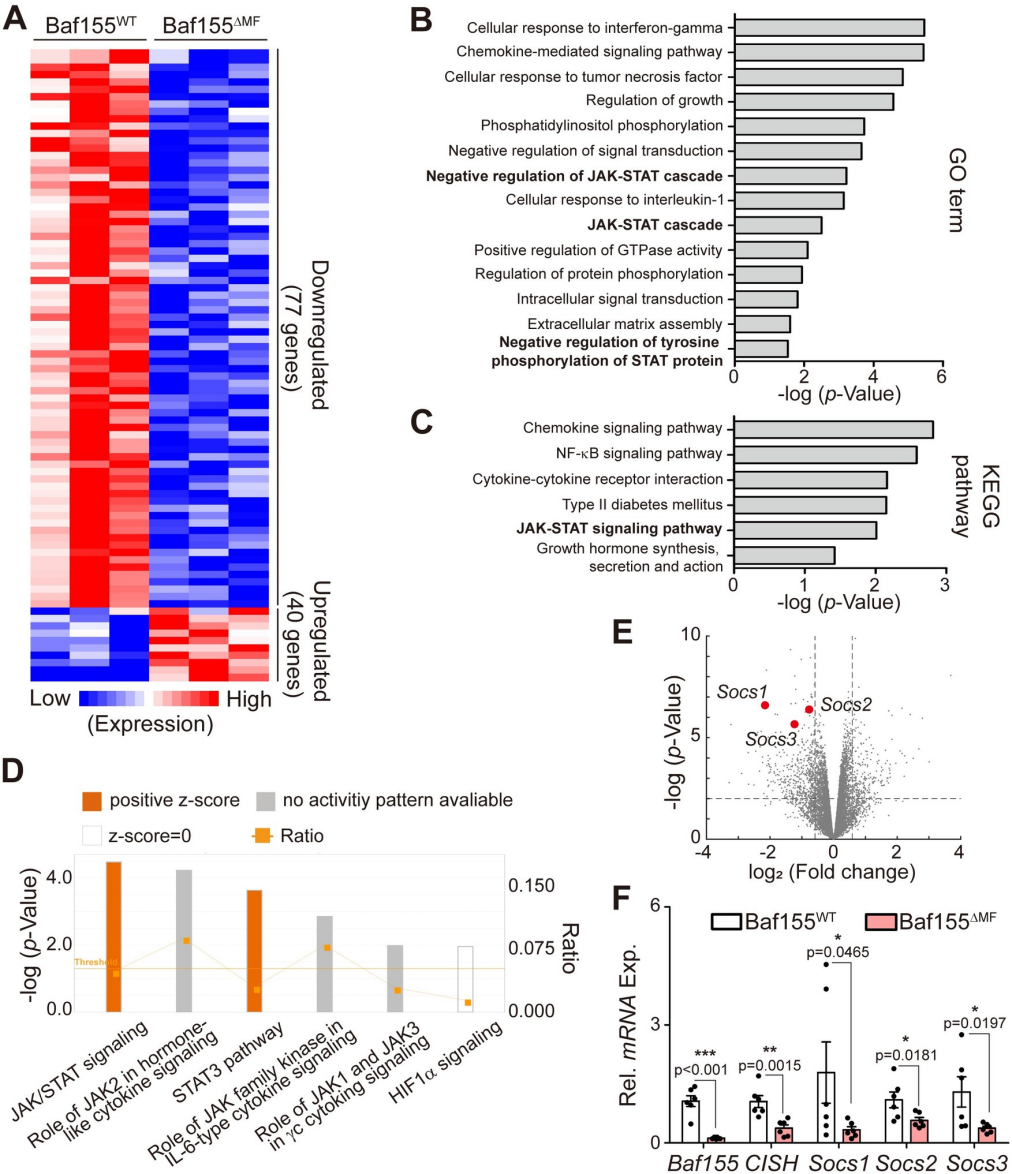
Baf155 ablation affects JAK/STAT signaling in skeletal muscle. **(A)** Heatmap of the DEGs in Baf155^WT^ and Baf155^ΔMF^ mice by RNA-seq using RNA from hind limb skeletal muscles (*n* = 3 mice per genotype). Each lane in the heatmap indicates each mouse (biological replicate). **(B)** The results from enrichment GO term analysis of DAVID Resources using the DEGs. JAK/STAT-related pathways were indicated by bold type. **(C)** The results from KEGG pathway enrichment analysis using the DEGs. JAK/STAT-related pathways were indicated by bold type. **(D)** Pathway enrichment in Baf155^ΔMF^ mice analyzed by IPA using the DEGs. The z-score predicts the direction of change for the function. The threshold was set to an absolute value of z-score = 2. The orange bars indicate the predicted inhibition, and the gray bars indicate that no activity prediction can be made by IPA. The ratio represents the number of molecules in DEGs that are involved in each indicated pathway divided by the total number of molecules that make up that pathway. **(E)** Volcano plot of DEGs in Baf155^ΔMF^ hind limb skeletal muscles compared to Baf155^WT^ hind limb skeletal muscles. Gray vertical lines partition the fold change (the left line for 0.75 fold, the right line for 1.5 fold), and gray horizontal line partition the *p*-value (*p* = 0.01). Each dot in the volcano plot represents 1 gene, and the red dots represent each indicated *SOCS* gene. **(F)** RT-qPCR analysis of *SOCS* genes in Q muscles of Baf155^WT^ and Baf155^ΔMF^ mice (*n* = 6 mice per each genotype). Each dot in the graph represents each mouse (biological replicate). Data are presented as mean ± SEM of biological replicates. Statistical analyses were performed using unpaired Student’s *t* test (*P < 0.05, **P < 0.01, ***P < 0.001 versus Baf155^WT^ control). The data underlying this figure can be found in S1 Data. Baf155, Brg1/Brm-associated factor 155; DEGs, differentially expressed genes; GO, gene ontology; IPA, ingenuity pathway analysis; JAK, janus kinase; KEGG, Kyoto encyclopedia of genes and genomes; MF, myofiber; Q, quadriceps; RT-qPCR, reverse transcription quantitative real-time PCR; SEM, standard error of the mean; SOCS, suppressor of cytokine signaling; STAT, signal transducer and activator of transcription.

To reveal the precise mechanism enhancing endurance exercise capacity in Baf155^ΔMF^ skeletal muscle, we performed mRNA-sequencing (RNA-seq) using hind limb skeletal muscles from unexercised mice and compared the transcriptomes between Baf155^WT^ and Baf155^ΔMF^ mice. A total of 117 genes, including 77 down-regulated and 40 up-regulated genes (<0.7-fold and >1.4-fold compared to Baf155^WT^ mice), were differentially expressed in Baf155^ΔMF^ mice (hereafter, DEGs) (Fig 3A). To identify interactions of DEGs, we performed Gene Ontology (GO) and Kyoto Encyclopedia of Genes and Genomes (KEGG) pathway analyses. Both analyses presented several biological processes by annotating DEGs and commonly detected inflammatory or immune response signaling and JAK-STAT signaling (Fig 3B and 3C and S1 and S2 Tables). We excluded inflammatory or immune response processes from the candidates due to the lack of relationship between the annotated genes, such as CCL9 and CCL21B, and skeletal muscle physiology [41,42]. To further analyze the interactions of DEGs, we performed an ingenuity pathway analysis (IPA). Similar to GO and KEGG pathway analyses, IPA also presented JAK/STAT3 signaling by annotating target genes and predicted the inhibition of this signaling in Baf155^ΔMF^ skeletal muscle (Fig 3D and S3 Table). In line with the inhibition of STAT3 signaling, which is the result of IPA, suppressors of cytokine signaling (SOCS) family (*CISH*, *SOCS1*, *SOCS2*, *SOCS3*) genes were down-regulated in Baf155^ΔMF^ skeletal muscle according to our RNA-seq data (Fig 3E), and we confirmed the reduced expressions of SOCS genes in Baf155^ΔMF^ skeletal muscle by RT-qPCR (Fig 3F). Together, these results revealed the inhibition of JAK/STAT3 signaling in Baf155^ΔMF^ skeletal muscle and suggested that the inhibition of this signaling could be related to the enhancement of endurance exercise capacity.

**Fig 4:**
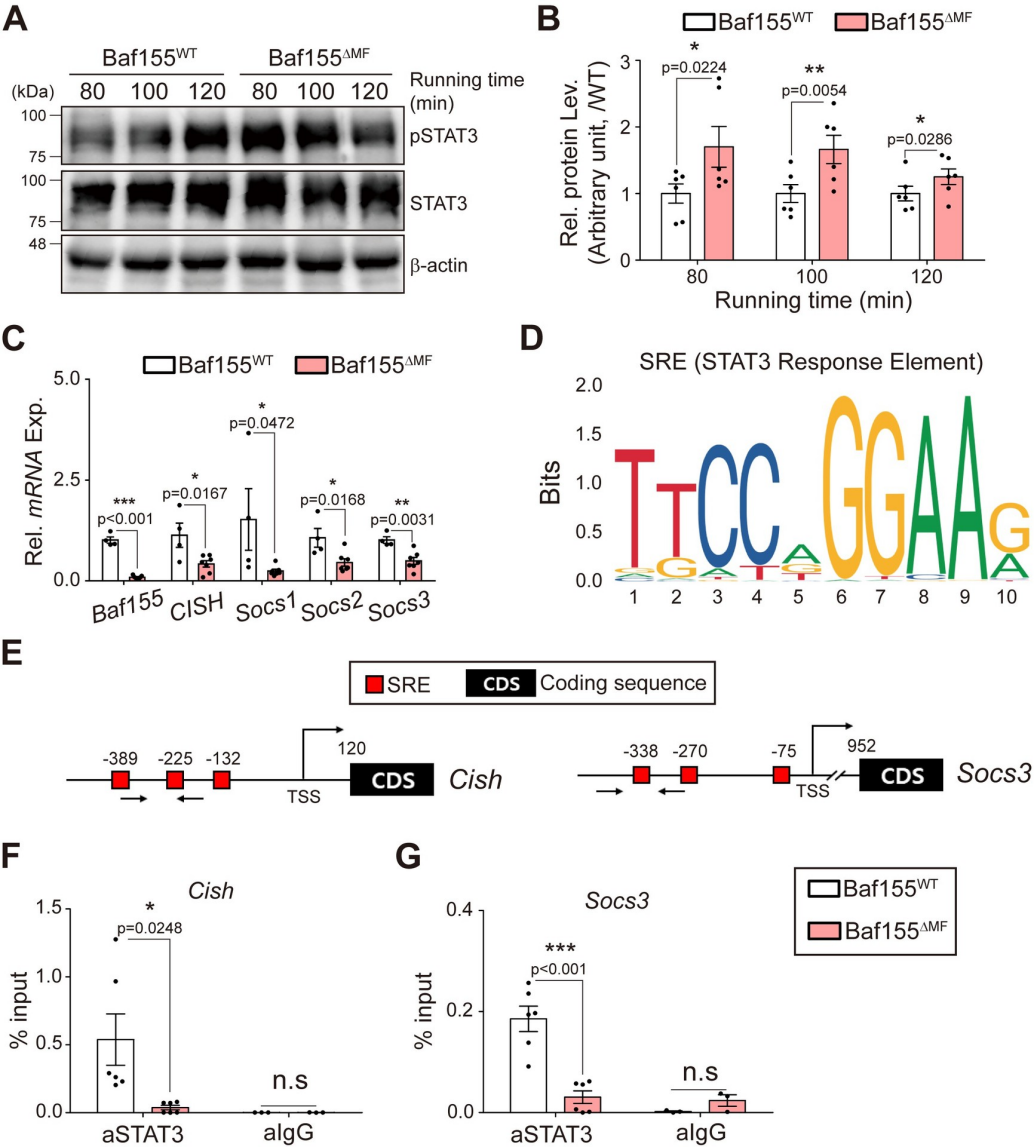
JAK/STAT signaling is inhibited due to the reduced DNA binding of STAT3. **(A)** Representative immunoblotting analyses of pSTAT3 and STAT3 in Q muscle of Baf155^WT^ and Baf155^ΔMF^ mice at each indicated time of treadmill running. Each lane in the immunoblotting image indicates each mouse. **(B)** The densitometric quantification of relative protein level of pSTAT3 in Q muscle of Baf155^ΔMF^ mice compared to Baf155^WT^ mice at the indicated time of treadmill running. The pSTAT3 level, which is normalized to STAT3, was used for the comparison. Six biological replicates of (A) were performed and quantified (*n* = 6 mice per each genotype at each indicated running time). **(C)** RT-qPCR analysis of *SOCS* genes in Q muscles of Baf155^WT^ and Baf155^ΔMF^ mice at 100 min of treadmill running (*n* = 4–7 mice per each genotype). **(D, E)** Prediction of STAT3-binding motif in the promoters of SOCS genes. The binding motif of STAT3 predicted by JASPAR (http://jaspar.genereg.net/) (D) and schematic representation of STAT3 binding motif positions in the promoter of each indicated gene. The number above symbol indicates the nucleotide length from TSS of each gene, and the arrow under the box indicates a primer binding site for ChIP-qPCR (E). **(F, G)** ChIP-qPCR analysis of STAT3 on the promoter of each indicated gene in Q muscle of Baf155^WT^ and Baf155^ΔMF^ mice at 100 min of treadmill running (*n* = 6 mice per genotype for aSTAT3, and *n* = 3 mice per genotype for aIgG). Each dot in the graphs (B, C, F, and G) represents each mouse (biological replicate). Data are presented as mean ± SEM of biological replicates. Statistical analyses were performed using unpaired Student’s *t* test (n.s., not significant; *P < 0.05; **P < 0.01; ***P < 0.001 versus Baf155^WT^ control). The data underlying this figure can be found in S1 Data. Baf155, Brg1/Brm-associated factor 155; ChIP-qPCR, chromatin immunoprecipitation-quantitative real-time PCR; IgG, Immunoglobulin G; JAK, janus kinase; MF, myofiber; Q, quadriceps; RT-qPCR, reverse transcription quantitative real-time PCR; SEM, standard error of the mean; SOCS, suppressor of cytokine signaling; STAT3, signal transducer and activator of transcription 3; TSS, transcription start site.

To verify this possibility, we analyzed STAT3 signaling in Baf155^ΔMF^ skeletal muscle during treadmill running. At a protein level, phospho-STAT3-Tyr705 (hereafter, pSTAT3), which is the active form of STAT3 and binds to DNA, was increased in Baf155^ΔMF^ skeletal muscle by 1.70, 1.66, and 1.25 folds compared to Baf155^WT^ skeletal muscle after running for 80, 100, and 120 min, respectively (Fig 4A and 4B). However, in contrast to the level of pSTAT3, SOCS genes were down-regulated in Baf155^ΔMF^ skeletal muscle (Fig 4C). Given that SWI/SNF complex is a chromatin remodeler [14], we assumed that the down-regulation of SOCS genes, despite the augmented pSTAT3, is due to the disturbance in DNA binding of pSTAT3 by Baf155 ablation. To verify this assumption, we investigated the bindings of pSTAT3 to its potential binding sites (STAT3 response elements, SREs) in promoters of SOCS genes [43] (Fig 4D and 4E) by performing ChIP analysis. As expected, the SRE bindings of pSTAT3 were diminished in Baf155^ΔMF^ skeletal muscle (Fig 4F and 4G). These results indicated that the impaired DNA binding of pSTAT3 reduced target gene expressions in Baf155^ΔMF^ skeletal muscle. Furthermore, considering SOCS genes are negative regulators of STAT3 signaling [44], our results implied that STAT3 phosphorylation was increased due to the down-regulation of SOCS. Together, our observations revealed that Baf155 is indispensable for the DNA binding of pSTAT3.

### Direct interaction of Baf155 with STAT3 contributes to STAT3 signaling in skeletal muscle

**Fig 5:**
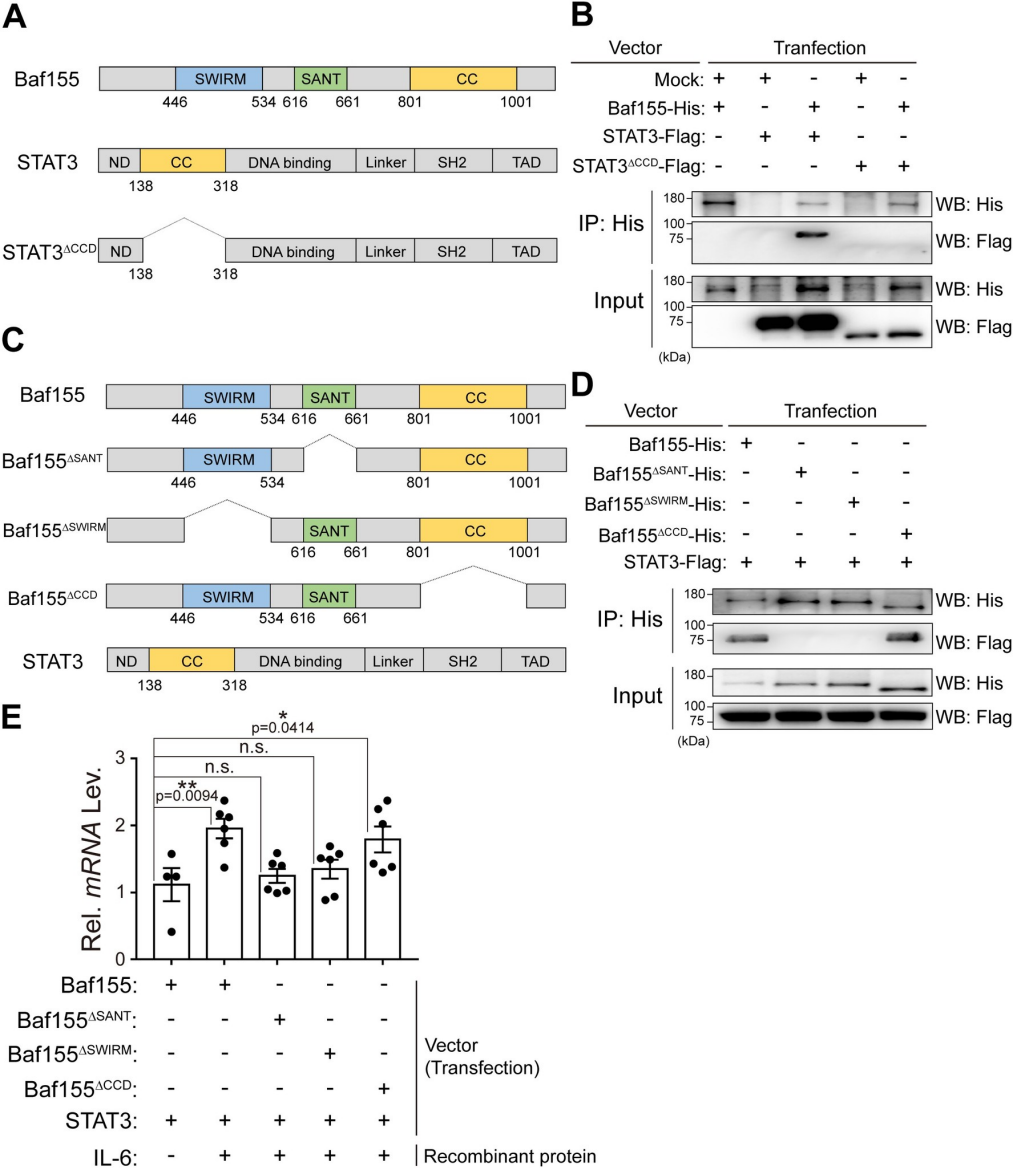
Direct interaction of Baf155 with STAT3 contributes to STAT3 signaling in skeletal muscle. **(A)** Schematic representation of domains of each indicated protein. The number under the domain indicates an amino acid number in each indicated protein. **(B)** Immunoblotting analysis of Co-IP of Baf155 with STAT3 or STAT3^ΔCCD^. Vector encoding Baf155-His, STAT3-Flag, or STAT3^ΔCCD^ was transfected, and each transfection condition was described above the immunoblotting image. A mock vector was used to adjust the total amount of DNA. **(C)** Schematic representation of domains of each indicated protein. The number under the domain indicates an amino acid number in each indicated protein. **(D)** Immunoblotting analysis of Co-IP of Baf155, Baf155^ΔSANT^, Baf155^ΔSWIRM^, or Baf155^ΔCCD^ with STAT3. Vector encoding Baf155-His, Baf155^ΔSANT^-His, Baf155^ΔSWIRM^-His, Baf155^ΔCCD^-His, or STAT3-Flag was transfected, and each transfection condition was described above the immunoblotting image. **(E)** RT-qPCR analysis of *CISH* expression in response to IL-6. Vector encoding Baf155, Baf155^ΔSANT^, Baf155^ΔSWIRM^, Baf155^ΔCCD^, or STAT3 was transfected. Each transfection and IL-6 treatment condition was described under the X-axis of the graph. Four or 6 biological replicates of each condition were performed, and each dot in the graph represents each biological replicate. Data are presented as mean ± SEM of biological replicates. Statistical analysis was performed using a one-way ANOVA test followed by Dunnett’s multiple comparison test (n.s., not significant; *P < 0.05, **P < 0.01, versus Baf155^+^ STAT3^+^IL6^−^ condition). The data underlying this figure can be found in S1 Data. Baf155, Brg1/Brm-associated factor 155; CCD, coiled-coil domain; CISH, cytokine-inducible SH2 containing protein; Co-IP, Co-immunoprecipitation; RT-qPCR, reverse transcription quantitative real-time PCR; SEM, standard error of the mean; STAT3, signal transducer and activator of transcription 3.

Since our results showed the requirement of Baf155 for the DNA binding of STAT3, we investigated the direct interaction between Baf155 and STAT3 by conducting Co-IP experiments. Since the coiled-coil domain (CCD) is known to mediate protein–protein interactions [45,46], we generated STAT3 without CCD (STAT3^ΔCCD^) (Fig 5A) and compared the interactions between Baf155 and STAT3 or STAT3^ΔCCD^. STAT3, but not STAT3^ΔCCD^, was precipitated along with Baf155 (Fig 5B). From this result, we concluded that Baf155 directly interacts with STAT3 and that the CCD of STAT3 is necessary for its interaction with Baf155. Next, we generated Baf155 without SANT, SWIRM, or CCD (Baf155 ^ΔSANT^, Baf155^ΔSWIRM^, or Baf155^ΔCCD^, respectively) to investigate the requirement of each domain for its interaction with STAT3 (Fig 5C). STAT3 was precipitated with Baf155 and Baf155^ΔCCD^ but not with Baf155^ΔSANT^ and Baf155^ΔSWIRM^ (Fig 5D). This result indicated that the SANT and SWIRM domains of Baf155 contribute to the interaction with STAT3. In addition, Baf155 ^ΔSANT^ and Baf155^ΔSWIRM^ attenuated the expression of STAT3 target gene compared to Baf155 (Fig 5E). This result indicated that the function of STAT3 requires interaction with Baf155. Together, our observations revealed that Baf155 contributes to STAT3 signaling by direct interaction with STAT3.

### Impaired DNA binding of pSTAT3 affects the function of HIF-1α in Baf155^ΔMF^ skeletal muscle

**Fig 6:**
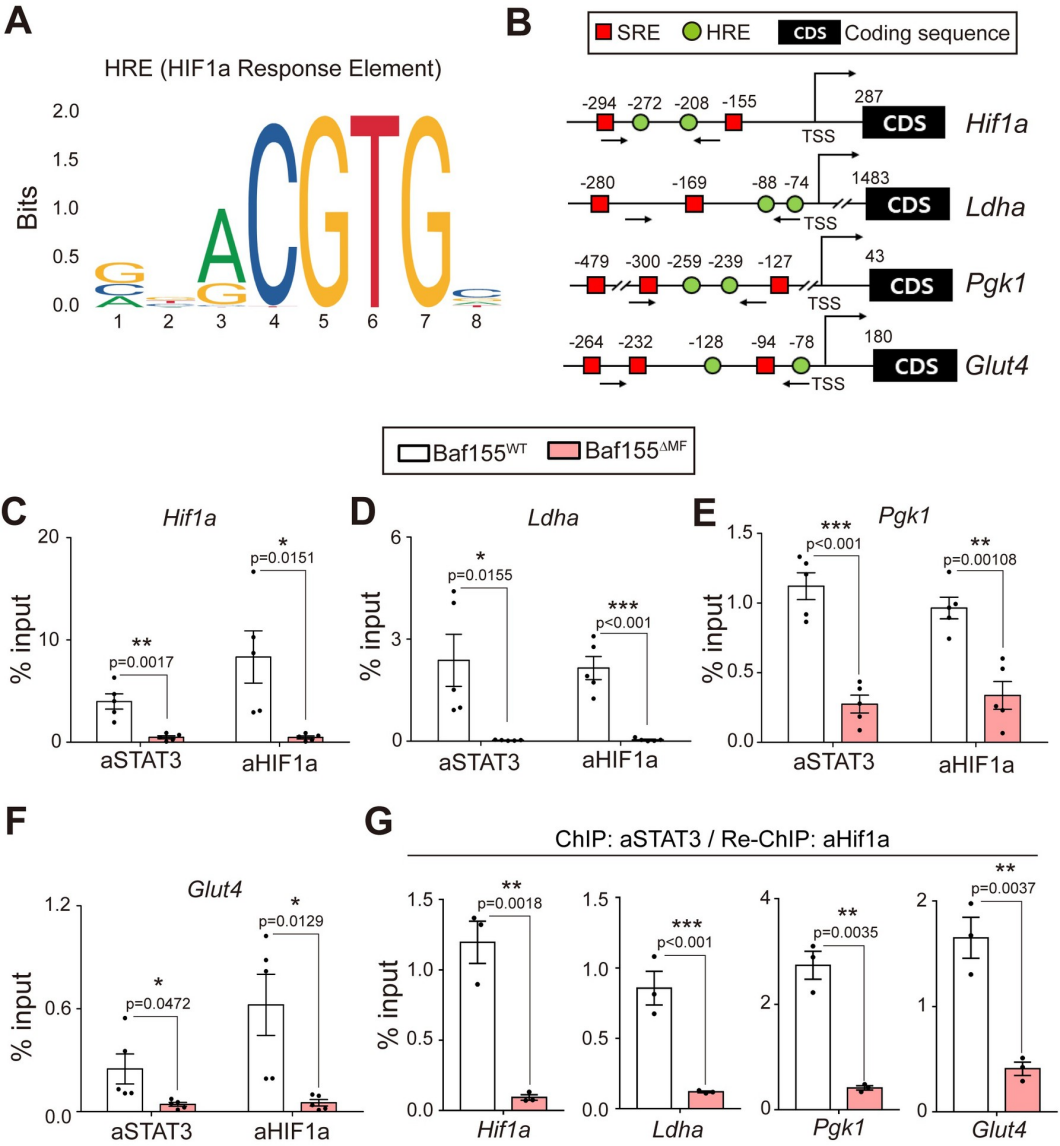
Impaired DNA binding of pSTAT3 affects the function of HIF-1α in Baf155^ΔMF^ skeletal muscle. **(A, B)** Prediction of STAT3-binding and HIF-1α-binding motifs in the promoters of HIF-1α target genes. The binding motifs of each transcription factor are predicted by JASPAR (http://jaspar.genereg.net/) (A) and schematic representation of binding-motif positions for each transcription factor in the promoter of indicated genes. The number above symbol indicates the nucleotide length from TSS of each gene, and the arrow under the box indicates the primer binding site for ChIP-qPCR (B). **(C–F)** ChIP-qPCR analyses of STAT3 and HIF-1α on the promoter of each indicated gene in Q muscle of Baf155^WT^ and Baf155^ΔMF^ mice at 100 min of treadmill running (*n* = 5 mice per each genotype). **(G)** ChIP-Re-ChIP qPCR analyses targeting the promoter of each indicated gene. The first round of ChIP used STAT3 antibody, and the second round of ChIP (Re-ChIP) used HIF1a antibody (*n* = 3 mice per each genotype). Primer sets, described in (B), were used for ChIP-Re-ChIP analyses. Each dot in the graphs (C, D, E, F, and G) represents each mouse (biological replicate). Data are presented as mean ± SEM of biological replicates. Statistical analyses were performed using unpaired Student’s *t* test (n.s., not significant; *P < 0.05; **P < 0.01; ***P < 0.001 versus Baf155^WT^ control). The data underlying this figure can be found in S1 Data. Baf155, Brg1/Brm-associated factor 155; ChIP-qPCR, chromatin immunoprecipitation-quantitative real-time PCR; HIF-1α, hypoxia-inducible factor-1α; MF, myofiber; Q, quadriceps; SEM, standard error of the mean; STAT3, signal transducer and activator of transcription 3; TSS, transcription start site.

Previous studies reported that pSTAT3 is essential for HIF-1α signaling due to mediating DNA binding of HIF-1α by forming the transcriptionally active complex [47–50]. HIF-1α signaling regulates energy metabolisms closely associated with exercise capacity [10–13]. Moreover, loss of HIF-1α in skeletal muscle enhances endurance exercise capacity by increasing oxidative metabolism [7]. Since the impairment of pSTAT3 function was observed concomitantly with the enhanced endurance exercise capacity in Baf155^ΔMF^ skeletal muscle, we hypothesized that the disruption of DNA binding of pSTAT3 enhances the endurance exercise capacity by reducing HIF-1α signaling. To verify this hypothesis, we first examined whether the DNA binding of HIF-1α is affected in Baf155^ΔMF^ skeletal muscle. We searched potential binding sites of HIF-1α, hypoxia response elements (HREs) (Fig 6A), and SREs (Fig 4D), which are close to the HREs, in promoters of HIF-1α target genes [43] (Fig 6B). Using ChIP analysis, we investigated the bindings of pSTAT3 and HIF-1α to each binding site. As SREs in SOCS gene promoters, the bindings of pSTAT3 to SREs in promoters of HIF-1α target genes were also disturbed in Baf155^ΔMF^ skeletal muscle (Fig 6C–6F). Besides, the binding of HIF-1α to HREs, adjacent to SREs, was also significantly diminished in Baf155^ΔMF^ skeletal muscle (Fig 6C–6F). These results indicated that Baf155 ablation resulted in the decreased DNA binding of HIF-1α to HREs in the promoters of target genes due to the impaired DNA binding of pSTAT3. To verify the association between HIF-1α and STAT3 in the promoters of target genes, we conducted ChIP-Re-ChIP analysis; STAT3 antibody for ChIP and HIF-1α antibody for Re-ChIP. The promoter regions of HIF-1α target genes, which were pulled down by the STAT3 antibody in the first round of ChIP, were pulled down again by the HIF-1α antibody in the second round of ChIP. However, this was significantly diminished in Baf155^ΔMF^ skeletal muscle. These results showed that HIF-1α interacts with STAT3 within the promoter regions of its target genes and indicated that Baf155 is involved in the interaction between STAT3 and HIF-1α within the promoter regions of HIF-1α target genes (Fig 6G). Furthermore, these results suggested that the reduced HIF-1α function could be associated with the enhanced endurance exercise capacity of Baf155^ΔMF^ skeletal muscle.

### Baf155 contributes to the DNA binding of the SWI/SNF complex to the promoters of STAT3 and HIF-1α target genes

**Fig 7:**
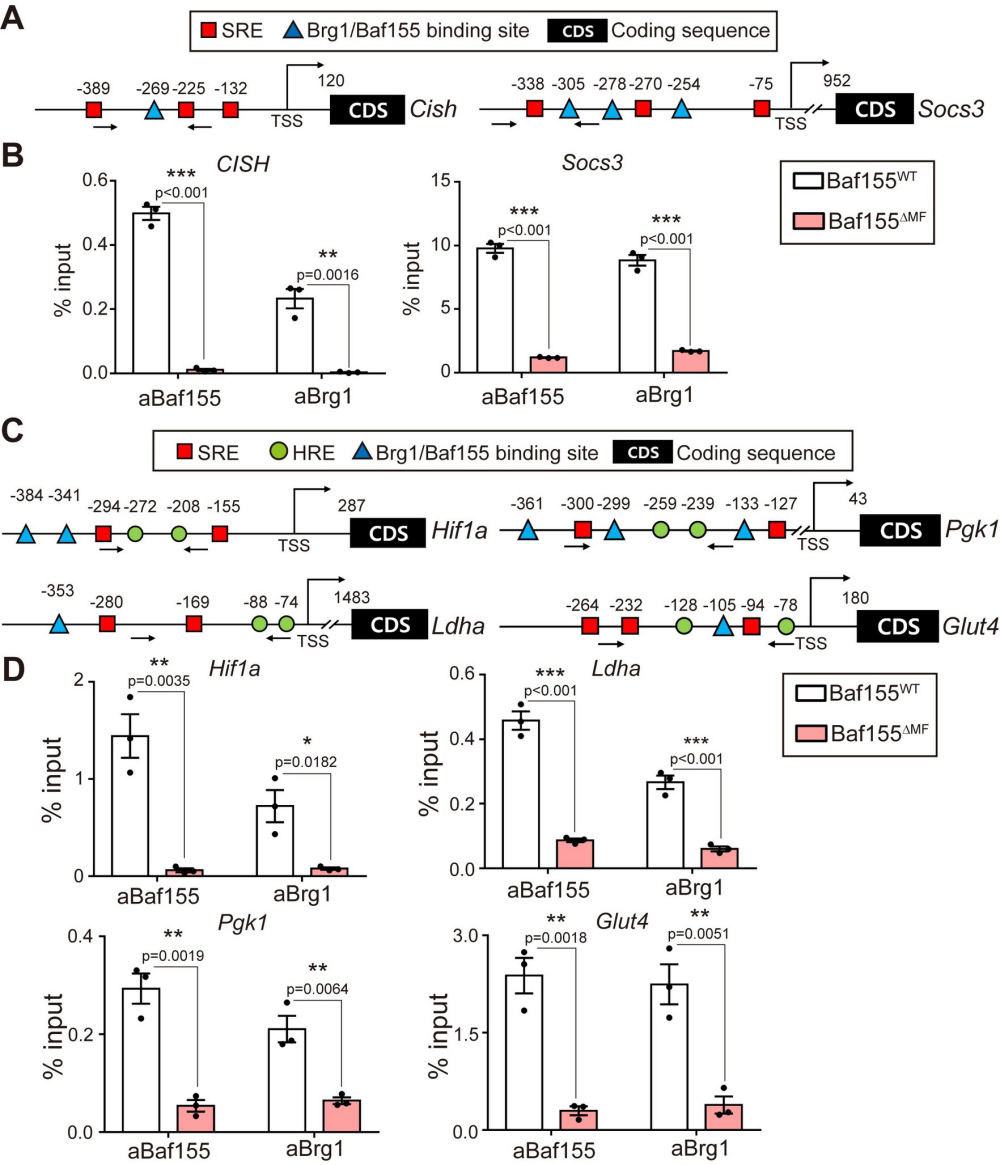
Baf155 contributes to the DNA binding of the SWI/SNF complex to the promoters of STAT3 and HIF-1α target genes. **(A)** Schematic representation of binding motif positions for STAT3, Baf155, and Brg1 in the promoter of indicated genes. **(B)** ChIP-qPCR analyses of Baf155 and Brg1 on the promoter of each indicated gene in Q muscle of Baf155^WT^ and Baf155^ΔMF^ mice after 100 min of treadmill running (*n* = 3 mice per each genotype). **(C)** Schematic representation of binding motif positions for STAT3, HIF-1α, Baf155, and Brg1 in the promoter of indicated genes. **(D)** ChIP-qPCR analyses of Baf155 and Brg1 on the promoter of each indicated gene in Q muscle of Baf155^WT^ and Baf155^ΔMF^ mice after 100 min of treadmill running (*n* = 3 mice per each genotype). The number above symbol indicates nucleotide length from the TSS of each gene, and the arrow under the site indicates the primer binding site for ChIP-qPCR (A and C). Each dot in the graph (B and D) represents each mouse (biological replicate). Data are presented as mean ± SEM of biological replicates. Statistical analyses were performed using unpaired Student’s *t* test (*P < 0.05, **P < 0.01, ***P < 0.001 versus Baf155^WT^ control). The data underlying this figure can be found in S1 Data. Baf155, Brg1/Brm-associated factor 155; Brg1, Brahma-related gene 1; ChIP-qPCR, chromatin immunoprecipitation-quantitative real-time PCR; HIF-1α, hypoxia inducible factor-1α; MF, myofiber; Q, quadriceps; SEM, standard error of the mean; STAT3, signal transducer and activator of transcription 3; TSS, transcription start site.

Next, we investigated the binding of Baf155 and Brg1 to the promoter regions of the target genes of STAT3 and HIF-1α. Since JASPAR provides only the binding sites of transcription factors, we could not use the database for the analysis of Baf155 and Brg1 binding sites, which are not transcription factors. Instead, we used ChIP-seq data from other studies for the prediction [51–53]. The predicted binding sites of Baf155 and Brg1 were close to the binding sites of STAT3 and HIF-1α in each indicated gene (Fig 7A and 7C). To validate the binding of Baf155 and Brg1 to predicted sites, we conducted ChIP-qPCR using primer sets that detect DNA bindings of STAT3 and HIF-1α (Figs 4E, 6B, 7A and 7C). Baf155 showed bindings to the predicted sites on the promoter regions of each indicated gene (Fig 7B and 7D). In addition, Brg1 showed reduced DNA bindings in Baf155 ^ΔMF^ skeletal muscle compared to Baf155^WT^ skeletal muscle (Fig 7B and 7D). These results indicated that Baf155 ablation affects the DNA binding of the SWI/SNF complex to the target genes. Together, our ChIP-qPCR analysis revealed the bindings of Baf155 and Brg1 to the promoter regions of the target genes of STAT3 and HIF-1α. Furthermore, our results showed the contribution of Baf155 to the DNA binding of the SWI/SNF complex on those sites.

### Reduction of HIF-1α signaling enhances the endurance exercise capacity by increasing oxidation

**Fig 8:**
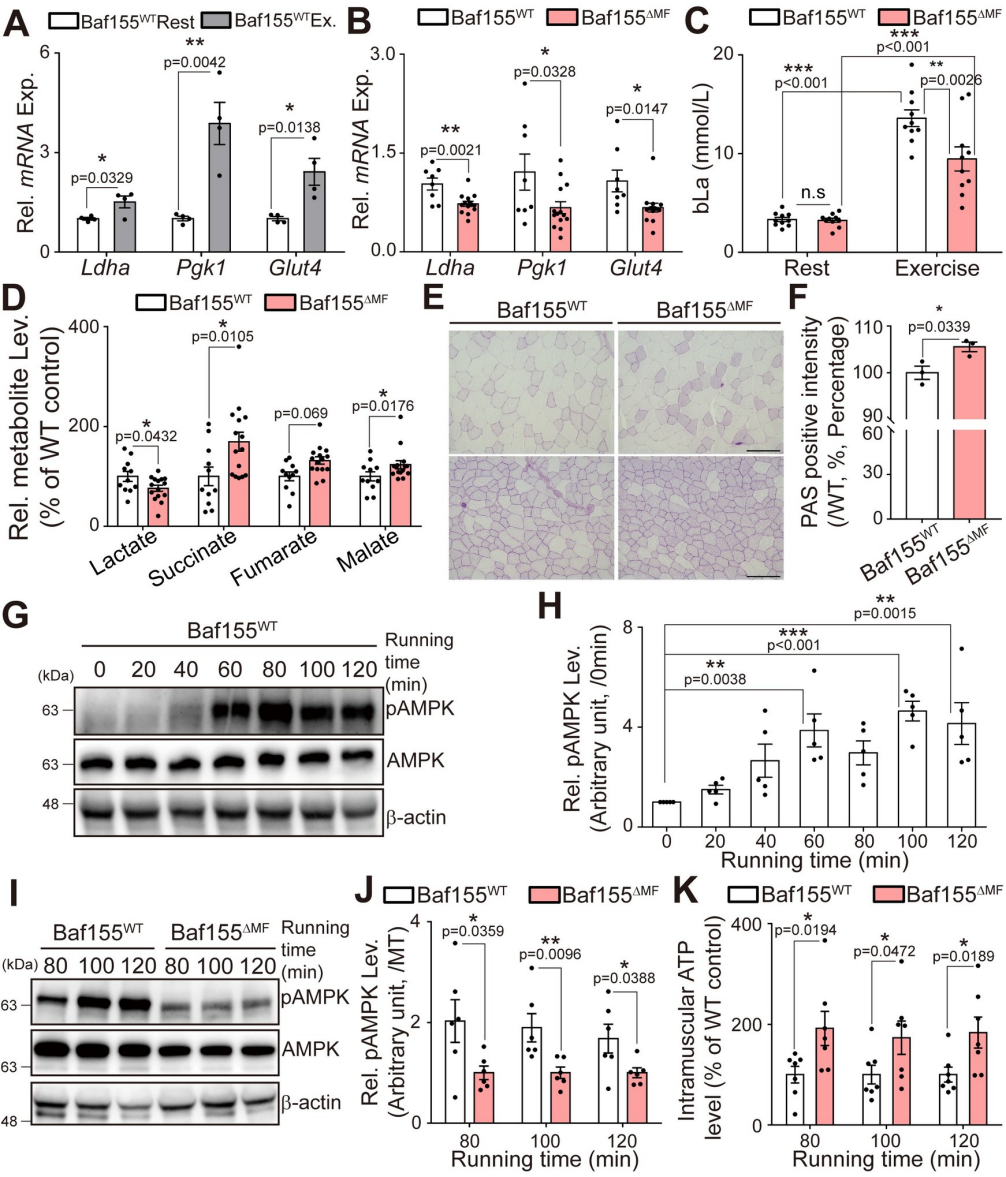
Reduction of HIF-1α signaling enhances the endurance exercise capacity by increasing oxidation. **(A)** RT-qPCR analysis of HIF-1α target genes in Q muscle of Baf155^WT^ mice at rest state and after 100 min of treadmill running (*n* = 4 mice per each condition). **(B)** RT-qPCR analysis of HIF-1α target genes in Q muscle of Baf155^WT^ and Baf155^ΔMF^ mice after 100 min of treadmill running (*n* = 8–14 mice per each genotype). **(C)** Blood lactate concentration in Baf155^WT^ and Baf155^ΔMF^ mice at rest state and at the exhaustion of treadmill running (*n* = 10 mice per each genotype). Statistical analysis was performed using a two-way ANOVA test followed by Tukey’s multiple comparison test (n.s., not significant; **P < 0.01, ***P < 0.001). **(D)** Relative metabolite level in muscle at the exhaustion of treadmill running. Metabolites were extracted from freshly isolated Q muscles at the exhaustion of treadmill running. Each metabolite was normalized to the external standard, and normalized level of metabolite was used for the comparison (*n* = 11–15 mice per each genotype). **(E)** PAS staining image of TA muscles from Baf155^WT^ and Baf155^ΔMF^ mice after 120 min of treadmill running. **(F)** The quantification of relative staining intensity of the whole section. Three biological replicates of (E) were performed and quantified (*n* = 3 mice per each genotype). **(G, H)** Representative immunoblotting analyses of pAMPK and AMPK in Q muscle of Baf155^WT^ mice after each indicated time of treadmill running. Each lane in the immunoblotting image indicates each mouse (G). The densitometric quantification of relative protein level of pAMPK in Q muscle of Baf155^WT^ mice after the indicated time of treadmill running. The pAMPK level, which is normalized to AMPK, was used for the comparison. Five biological replicates of (G) were performed and quantified (*n* = 5 mice per each condition). Statistical analysis was performed using one-way ANOVA followed by Tukey’s multiple-comparisons test (**P < 0.01, ***P < 0.001 versus Baf155^WT^ mice at rest state) (H). **(I, J)** Representative immunoblotting analyses of pAMPK and AMPK in Q muscle of Baf155^WT^ and Baf155^ΔMF^ mice after each indicated time of treadmill running. Each lane in the immunoblotting image indicates each mouse (I). The densitometric quantification of relative protein level of pAMPK in Q muscle of Baf155^WT^ and Baf155^ΔMF^ mice after the indicated time of treadmill running. The pAMPK level, which is normalized to AMPK, was used for the comparison. Six biological replicates of (I) were performed and quantified (*n* = 6 mice per each genotype with indicated running time) (J)**. (K)** Luminometric analysis of intramuscular ATP level in Q muscle of Baf155^WT^ and Baf155^ΔMF^ mice after each indicated time of treadmill running (*n* = 7 mice per each genotype with indicated running time). Each dot in the graphs (A–D, F, H, J, and K) represents each mouse (biological replicate). Data are presented as mean ± SEM of biological replicates. Statistical analyses were performed using unpaired Student *t* test (*P < 0.05, **P < 0.01, ***P < 0.001 versus control) (A, B, D, F, J, and K). The data underlying this figure can be found in S1 Data. AMPK, 5′ adenosine monophosphate-activated protein kinase; ATP, adenosine triphosphate; Baf155, Brg1/Brm-associated factor 155; HIF-1α, hypoxia inducible factor-1α; MF, myofiber; PAS, periodic acid and Schiff; Q, quadriceps; RT-qPCR, reverse transcription quantitative real-time PCR; SEM, standard error of the mean.

Based on the reduction in DNA binding of HIF-1α in Baf155^ΔMF^ skeletal muscle, we next investigated expressions of its target genes. In Baf155^WT^ mice, target genes, which regulate glycolysis, were up-regulated after treadmill running (Fig 8A). However, the expressions of these genes were significantly reduced in Baf155^ΔMF^ mice compared to Baf155^WT^ mice after performing the same intensity of exercise (Fig 8B). This result indicated that HIF-1α signaling was reduced in Baf155^ΔMF^ skeletal muscle due to the impaired DNA binding of HIF-1α. As previously described, loss of HIF-1α signaling increases oxidation in skeletal muscle, which enhances endurance exercise capacity [7]. Lactate, the main product of glycolysis, is released from skeletal muscle into the bloodstream [54]. Moreover, increased oxidation in skeletal muscle enhances exercise capacity accompanying low blood lactate concentration (bLa) during exercise [10,54–57]. Considering these reports, we expected low bLa in Baf155 ^ΔMF^ mice due to the increased oxidation and measured bLa at the exhaustion of treadmill running. As expected, Baf155^ΔMF^ mice showed lower bLa even after running for a longer time and greater distance than Baf155^WT^ mice (Fig 8C). To furtherly analyze the intramuscular metabolism during exercise, we conducted a targeted metabolomics analysis at the exhaustion of treadmill running. Consistent with bLa, the intramuscular lactate level was significantly lower in Baf155^ΔMF^ skeletal muscle than in Baf155^WT^ skeletal muscle. Contrary to lactate, the metabolites of the aerobic metabolism, such as succinate, fumarate, and malate, were higher in Baf155^ΔMF^ skeletal muscle than in Baf155^WT^ skeletal muscle. These results showed a relatively high aerobic metabolism in Baf155^ΔMF^ skeletal muscle than in Baf155^WT^ control. Furthermore, these results implied that the intramuscular metabolism during exercise depends relatively more on the aerobic metabolism in Baf155^ΔMF^ mice than in Baf155^WT^ mice (Fig 8D). In line with increased oxidation, Baf155^ΔMF^ skeletal muscle showed a relatively higher intramuscular glycogen level compared to Baf155^WT^ skeletal muscle after performing the same exercise intensity (Fig 8E and 8F). These results indicated that the reduced HIF-1α signaling by the impaired DNA binding of HIF-1α led to higher oxidation in Baf155^ΔMF^ skeletal muscle during exercise.

Skeletal muscle requires ATP for a contraction, and oxidative metabolisms mainly provide ATP for skeletal muscle during long-term exercise [58]. Since Baf155^ΔMF^ mice showed higher oxidation than Baf155^WT^ mice, we expected a difference in intramuscular ATP level during exercise. To verify our expectation, we first examined the activation of adenosine monophosphate-activated protein kinase (AMPK), a hallmark of energy balance, in skeletal muscle [59–64]. Baf155^WT^ mice showed a significant increment of phospho-AMPK-Thr172 (hereafter, pAMPK) after running for more than 60 min (Fig 8G and 8H). Since an increase of [AMP or ADP]: [ATP] ratio determines the phosphorylation of AMPK, our observation indicated that ATP consumption rate exceeded its synthesis rate in Baf155^WT^ skeletal muscle after running for more than 60 min following our treadmill running scheme. However, interestingly, the level of pAMPK was significantly lower in Baf155^ΔMF^ skeletal muscle than in Baf155^WT^ skeletal muscle after performing the same intensity of exercise (Fig 8I and 8J). This result indicated that Baf155^ΔMF^ skeletal muscle retained a higher [AMP or ADP]: [ATP] ratio than Baf155^WT^ skeletal muscle during exercise. Based on this observation, we expected a higher ATP level in Baf155^ΔMF^ skeletal muscle and compared an intramuscular ATP level during exercise. In line with the decreased pAMPK level, Baf155^ΔMF^ skeletal muscle contained more ATP than Baf155^WT^ skeletal muscle after performing the same intensity of exercise (Fig 8K). This result implied that the enhanced endurance exercise of Baf155^ΔMF^ skeletal muscle is due to the high intramuscular ATP during exercise. Together, our observations revealed that the high oxidation due to the reduced HIF-1α signaling resulted in the increased intramuscular ATP level, which enhanced the endurance exercise capacity.

Since Baf155 ablation resulted in metabolic alterations, we examined whether Baf155^ΔMF^ mice are more prone to develop metabolic defects than Baf155^WT^ mice in response to high-fat diet feeding. We underwent high-fat feeding in Baf155^WT^ and Baf155^ΔMF^ mice for 8 weeks, a method previously described that sufficiently induces metabolic changes [65]. Body weight and weight gain percentage after high-fat feeding were comparable between Baf155^WT^ and Baf155^ΔMF^ mice (S4A and S4B Fig). In addition, masses of hind limb muscle, fat, and liver after high-fat feeding were also similar in both mouse models (S4C–S4E Fig). These results showed similar responses of Baf155^WT^ and Baf155^ΔMF^ mice to the high-fat diet. Furthermore, we conducted a glucose tolerance test (GTT). Blood glucose levels were similar in both mouse models under the normal chow condition and after high-fat diet feeding (S4F–S4I Fig). Altogether, these results revealed that Baf155^ΔMF^ mice are not susceptible to metabolic defects in response to a high-fat diet compared to Baf155^WT^ mice, despite the metabolic alteration in skeletal muscle during exercise.

## Discussion

The results of this study reveal the biological function of Baf155 in the energy metabolism of skeletal muscle, modulating hypoxia signaling. Albeit showing normal development and maturation of skeletal muscle, Baf155^ΔMF^ mice show a significant improvement in endurance exercise capacity. The ablation of Baf155 impedes glycolysis, resulting in decreased lactate production and increased intramuscular ATP production during endurance exercise, which leads to the enhancement of exercise capacity. The metabolic changes are rooted form the alteration of HIF-1α signaling, where Baf155 mediates this signaling by regulating the DNA binding of HIF-1α. Moreover, the regulatory function of Baf155 in HIF-1α signaling is associated with pSTAT3, which forms a coactivator complex with HIF-1α to fully activates HIF-1α signaling (S5 Fig).

Recent studies have revealed specific functions of Baf subunits by showing the interactions between Bafs and other transcription factors [66,67]. In skeletal muscle, for example, Baf47 regulates the terminal differentiation of muscle stem cells by interacting with a myogenic factor MyoD [18]. In addition, Baf60c determines myofiber types by interacting with the Six4 transcription factor [19]. Despite the discovery of new functions of other Bafs, Baf155 has been known as a structural protein stabilizing other subunits [22,23]. In the present study, we identified the function of Baf155 in skeletal muscle for the first time, to our knowledge. Our results showed that Baf155 regulates the energy metabolism of skeletal muscle via HIF-1α signaling. Furthermore, we also showed that Baf155 executes this function by mediating the DNA binding of HIF-1α through the interaction with pSTAT3. These results identify the new function of Baf155 in skeletal muscle, reveal the functional interaction between Baf155 and HIF-1α through pSTAT3, and suggest the possibility of unrevealed functions of Baf155 in other organs. In addition, considering the results of our ChIP-qPCR and Co-IP experiments, our observations reveal the contribution of Baf155 to the recruitment of the SWI/SNF complex, the DNA binding of transcription factors STAT3 and HIF-1a, and the expressions of target genes. Although the relationship between SWI/SNF complex recruitment, STAT3/HIF-1α DNA binding and target gene expression can be implied, the precise mechanism and causality underlying our observations could not be fully revealed in the present study. Further study is required to clarify the detailed mechanism for the function of Baf155 in skeletal muscle.

Previous studies reported the reduced stability of subunits by Baf155 ablation in in vitro (kidney-derived COS-1 cell) or in vivo (mouse thymus) experiments [22,68]. Mechanistically, Baf155 stabilizes other subunits by blocking the binding of E3 ubiquitin ligase, checkpoint with forkhead and ring finger domain (CHFR) [23]. However, depending on our observations, Baf155 ablation does not affect the stability of other Baf subunits in skeletal muscle. These results strongly suggest other biological mechanisms protecting Baf subunits independently of Baf155. For example, the compensatory role of other Bafs, such as Baf170, which is a paralogue having similar structure to Baf155 [69–71], or the muscle-specific mechanisms protecting subunits from proteasomal degradation could contribute to the maintenance of Baf subunits in skeletal muscle. Further study is essentially required to understand the stabilization of the SWI/SNF complex in skeletal muscle.

Skeletal muscle has remarkable plasticity responding to external stimuli [72–74], and different gene clusters show distinct patterns of expressions depending on the type of stimulus [75–77]. Contractile activity, such as exercise, also induces expressional changes in various gene clusters in skeletal muscle [34,78]. The effect of acute exercise returns to the pre-exercise state within hours after cessation, but long-term exercise elicits chronic changes accompanying the enhancement of exercise capacity [79,80]. In previous studies, researchers tried to find crucial genes related to the enhancement of exercise capacity by analyzing transcriptomes in skeletal muscle after long-term exercise [81–83]. Although these studies revealed the gene clusters showing expressional changes, researchers could not directly prove the relation between these genes and exercise capacity. Thus, further studies about the function of these genes in skeletal muscle are essential to reveal the molecular mechanism enhancing exercise capacity. Smarcc1, a homolog of Baf155 in humans, also shows an expressional change in skeletal muscle with enhanced exercise capacity [84]. Stepto and colleagues compared transcriptomes between healthy but untrained control subjects and well-trained athletes. They identified approximately 260 differentially expressed genes (DEGs) (161 up-regulated and 102 down-regulated), and Smarcc1 is one of the major down-regulated genes in the athlete’s skeletal muscle. Stepto and colleagues suggested that DEGs from their microarray experiments would include crucial genes contributing to the enhancement of exercise capacity but could not experimentally verify biological relations between these genes and the enhanced exercise capacity. Here, we show that a reduction in Baf155 enhances exercise capacity. Considering our results and the report from Stepto and colleagues, Baf155 could be one of the crucial genes contributing to the enhancement of exercise capacity in skeletal muscle. Furthermore, we also reveal the regulatory mechanism of Baf155 in the HIF-1α signaling activation. Given that the precise mechanisms enhancing the endurance exercise capacity are still not fully elucidated, our results improve biological understanding of the enhancement mechanism of exercise capacity in skeletal muscle and suggest Baf155 as a crucial gene for this process.

Moreover, we showed that HIF-1α signaling is impeded in Baf155 ablated skeletal muscle, strongly supporting our suggestion that Baf155 is a crucial gene for enhancing exercise capacity. Since HIF-1α regulates expressions of genes related to hypoxic response and is dramatically activated in exercising skeletal muscle [10,11,85], HIF-1α has been considered as beneficial for the maintenance of exercise capacity. Recent studies, however, revealed HIF-1α signaling inhibits oxidative metabolism [86–88] and the suppression of this signaling in skeletal muscle results in improved exercise capacity [89,90]. Furthermore, the ablation of HIF-1α in skeletal muscle improves endurance exercise capacity due to the decrement of glycolytic metabolism [7]. These results indicate that molecular mechanisms inhibiting HIF-1α signaling in skeletal muscle could improve exercise capacity. In this study, we showed that Baf155 is involved in HIF-1α signaling, and the ablation of Baf155 impedes this signaling in skeletal muscle. Depending on these results, we suggest that the down-regulation of Baf155 in skeletal muscle, leading to an impediment of HIF-1α signaling, is one of the molecular mechanisms enhancing exercise capacity.

Although showing the reduction of the glycolytic pathway in Baf155^ΔMF^ skeletal muscle, we could not fully reveal how the reduced glycolysis results in enhanced oxidative metabolism. According to previous reports, the partial pressure of oxygen (PO_2_) in skeletal muscle maintains above the required PO_2_ for mitochondrial function even during exercise [91–94]. Considering these results, pyruvate could be metabolized by the oxidative pathway or glycolytic pathway in exercising skeletal muscle. However, the increased enzymatic flux of LDHA, the key enzyme of the glycolytic pathway, during exercise leads pyruvate to the lactate generation pathway [95]. Altogether, the change of enzymatic flux of LDHA could be a possible mechanism underlying the metabolic alteration in Baf155 ^ΔMF^ skeletal muscle, but we could not verify this possibility. Further investigation of the enzymatic flux might reveal the precise mechanism for the altered metabolism in Baf155^ΔMF^ skeletal muscle.

Statisticians raise concerns about the interpretation of the *p*-value and suggest additional statistical analyses that could supplement the *p*-value. They propose that presenting other statistical analyses in addition to the *p*-value would strengthen the statistical significance of the evidence [96–100]. The calculation of false positive risk (FPR) is one of the suggested statistical analyses and quantifies the probability of the experimental results [100]. Considering this, we calculated the FPRs of the data with a one-star *p*-value (0.01<*p*<0.05) (S6 Table). Our data showed FPRs under 20%, and this numerical value is less than the rejection threshold, which means the statistically suggestive depending on the FPR [100,101]. However, the standard for the threshold to decide the statistical efficiency of data is still controversial. In line with this, statisticians propose the requirement of considering the statistical threshold depending on research fields and suggest methods to calculate the statistical basis for thresholds [102–105]. We believe that biologists also have to discuss the standard of a threshold of *p*-value, FPR, or other statistical analyses to achieve statistical improvement in biology, and this will strengthen the statistical significance of biological discoveries.

In summary, our study identifies Baf155 as a regulator of energy metabolism in the skeletal muscle and shows that Baf155 regulates HIF-1α signaling by mediating its DNA binding. In line with this fundamental role, the ablation of Baf155 impedes the HIF-1α signaling, results in the altered energy metabolism in skeletal muscle, and consequently enhances the endurance exercise capacity. These results reveal the relationship between Baf155 and exercise physiology while suggesting that modulation of Baf155 function could be a strategic target for the enhancement of energy metabolism and exercise capacity in skeletal muscle.

## Materials and methods

### Ethics statement

All animal experiments followed the Laboratory Animal Act and the Animal Protection Act of South Korea. The animal experiments were approved by and under the post-approval monitoring of the Institutional Animal Care and Use Committee (IACUC) of the Seoul National University (SNU-191231-2).

### Animals

All male mice (C57BL/6J background) were housed in a specific pathogen-free animal facility, maintained on a 12 h light-dark cycle, and fed with normal chow unless otherwise indicated. Mice carrying floxed *Baf155* were provided by Dr. Seong RH (Department of Biological Sciences, Seoul National Univ., Seoul, Korea). *MCK*-Cre transgenic mice and *Myh6*-MerCreMer transgenic mice were purchased from The Jackson Laboratory. Mouse studies were performed according to the guidelines of the ethical committees at Seoul National University. All exercise function tests were performed at a fixed time of the day (3 PM) and in the same place. In addition, GTT and blood lactate measurement tests were also performed in the aforementioned restricted spatiotemporal exercise condition to exclude the effects of environmental changes or circadian rhythms. For the ablation of Baf155 in cardiac muscle, tamoxifen (20 mg/mL in corn oil) (Sigma-Aldrich, St. Louis, Missouri, United States of America) was administered orally to Baf155^f/f^ or *Myh6*-MerCreMer; *Baf155*^f/f^ for 5 consecutive days (160 mg/kg body weight).

### Four limb grip strength test

Grip strength was evaluated using a grip strength test meter (grip strength test BIO-GS3, Bioseb). Mice were allowed to grasp an attached grid with 4 limbs and were horizontally pulled by the tip of tail. The test was performed for at least 5 times with 10 min of resting between each measurement. The average of the top 3 grip strength (N, Newton) was normalized to body weight (g) (N/g). All experiments were performed in a blind fashion.

### Inverted grid hanging test

Mice were placed on the center of a grid (20 × 20 cm, consisting of 1.2 cm squares of 0.1 cm diameter wire) mounted 30 cm above a padded surface. A weight about 10% body weight of each mouse was suspended from a tail by a clip to avoid the hanging with tails. The grid was inverted and latency to fall from the grid was recorded. Mice performed hanging test for at least 4 trials with 30 min of resting between each trial. The average of latency to fall (min) was normalized to body weight (min*g). All experiments were performed in a blind fashion.

### Treadmill running test

Mice were acclimated to the treadmill (DJ2-242 Dual Treadmill, Daejong Instrument, Treadmill for 8 mice LT320, Maze engineers) before running tests. The acclimation scheme was the 10-min running at a speed of 10 m/min for 3 consecutive days. After the training period, on the fourth day, Baf155^WT^ and Baf155^ΔMF^ mice were allowed to run until exhaustion for endurance function tests. Running speed was set to 10 m/min for 30 min and increased by 2 m/min every 20 min with no inclination. Exhaustion was determined by the inability of the mice to run on the treadmill more than 10 s despite stimulation. All experiments were performed in a blind fashion. For analysis of RNA, protein, or ATP, Baf155^WT^ and Baf155^ΔMF^ mice performed the same intensity of running with pre-described running protocol and stopped at the appointed time. Mice were euthanized right after running, and freshly isolated skeletal muscles were processed with respective methods for each analysis.

### Reverse transcription quantitative PCR (RT-qPCR)

Total RNA was extracted from freshly isolated hind limb muscles from Baf155^WT^ and Baf155 ^ΔMF^ mice using TRIzol reagent (Invitrogen) and 2 μg of total RNA was reverse transcribed using RT system (ImProm-II reverse transcription system, Promega). A 1/30 dilution of complementary DNA was used to assess gene expression by SYBR Green technology (TB Green Premix Ex Taq-Tli RNaseH Plus, Takara). Relative expression levels of genes were calculated by 2^−ΔΔC^_T_ (C_T_; threshold cycle), ΔΔC_T_ means the difference of C_T_ between target genes and β-actin (reference gene). C_T_ was analyzed by Rotor-Gene Q software (QIAGEN). Primer sequences are appended in S4 Table.

### mRNA sequencing (RNA-seq) and bioinformatics analyses

Total RNA was extracted from freshly isolated hind limb muscles from unexercised Baf155^WT^ and Baf155^ΔMF^ mice, respectively, using TRIzol reagent (Invitrogen). To avoid biased analysis induced by specificity of particular skeletal muscles, we used whole hind limb muscles (TA, EDL, GA, SOL, and Q) for RNA extraction. The RNA-seq library was generated by TruSeq Stranded Total RNA LT sample Prep Kit (Illumina) and sequenced with the HiSeq 2500 Illumina genome sequencer. DEG list from RNA-seq was used for further bioinformatic analyses. GO and KEGG pathway analyses database (https://david.ncifcrf.gov/) was used for identifying enriched GO terms in Baf155^ΔMF^ skeletal muscle. Bioinformatic evaluation of DEGs in Baf155^ΔMF^ skeletal muscle was performed by IPA (http://www.qiagenbioinformatics.com, QIAGEN). The raw RNA-seq dataset is deposited on the Gene Expression Omnibus (GEO, https://www.ncbi.nlm.nih.gov/geo/query/acc.cgi) with the accession number of GSE 163373.

### Western blot analysis

The following primary antibodies were used in this study: mouse anti-pAMPKα (Thr172) (#2535), AMPKα (#2532), STAT3 (#9132), and Gapdh (#2118) from Cell Signaling; Baf60b (sc-101162), Baf170 (sc-17838) from Santa-Cruz; rabbit anti-β-actin (A2066) from Sigma-Aldrich, Baf60a (#35070), Baf60c (#62265), pSTAT3 (Tyr705) (#9145) from Cell Signaling; Arid1a (ab182560), Baf155 (ab172638), Brm (ab240648), Brg1 (ab110641) from Abcam. The following secondary antibodies appropriating for each primary antibody were used: HRP-conjugated anti-Mouse IgG (w4021) and HRP-conjugated anti-rabbit IgG (w4011) from Promega. All primary antibodies were diluted 1:1,000 with TBS containing 0.1% Tween-20 and 5% BSA. Protein lysates were extracted from freshly isolated quadriceps using RIPA buffer mixed with 1× protease inhibitor (Halt protease inhibitor cocktail, Thermo Scientific), phosphatase inhibitor (Phosphatase inhibitor cocktail 3, Sigma-Aldrich), and pepstatin (1 μg/mL). Bradford’s reagent (Bio-Rad Laboratories) was used for estimating protein concentration. Proteins were separated by 10% polyacrylamide gels and transferred to PVDF membranes (Millipore). Membranes were blocked with 5% BSA or 5% skim milk for 2 h at room temperature (RT) and incubated with respective primary antibodies at 4°C for overnight. After incubation with secondary antibodies, membranes were developed with SuperSignal West Dura Extended Duration substrate (Thermo Scientific) and visualized with a Fusion solo chemi-luminescence imaging system (Vilber Lourmat). Densitometric analysis of immunoblot data was performed with ImageJ software (NIH).

### Histological analysis

Skeletal muscle samples were immediately embedded in optimal cutting temperature (OCT) compound (SAKURA) after dissection, frozen with liquid nitrogen, and stored at −80°C till analysis. IHC using muscle cryosections were performed by following procedures: For muscle stem cell staining, 7 μm sections were dried at RT for 10 min, fixed with 4% paraformaldehyde for 10 min, washed with PBS, and performed antigen retrieval using citrate buffer (0.01 M citrate in distilled water, pH 6.0). The slides were treated with blocking reagent (M.O.M. blocking reagent, Vector Laboratories) following the recommended protocol and incubated with respective primary antibodies for overnight at 4°C. After washing with PBS, the slides were incubated with the appropriate secondary antibodies at RT for 1 h, counterstained for nuclei with Hoechst (Invitrogen), and mounted with Vectashield (Vector Laboratories). For myosin heavy chain (MyHC) staining, unfixed sections were used for staining and followed the protocol same with stem cell staining except for antigen retrieval. Stained sections were visualized with a fluorescent microscope (Axio observer Z1, Zeiss) and analyzing software (Leopard, ZOOTOS) were used for cross-sectional area (CSA) analysis. For NADH staining, air-dried cryosection of skeletal muscle was incubated in staining solution (MTT 0.25 mg, 50 mM Tris-HCl (pH 7.4), 5 mM MgCl2, 25 mM CoCl2 (pH 7.0), 2 mg Co-enzyme NADH) for 90 min at 37°C. After incubation, section was fixed in 4% PFA for 15 min and washed with DW, followed by mount with aqueous mounting medium. For PAS staining, cryosections of skeletal muscle was fixed using 4% paraformaldehyde for 10 min at RT followed by washing with PBS. The slides were oxidized in 0.5% periodic acid solution for 5 min, rinsed in distilled water for 3 times, and incubated in Schiff’s reagent for 15 min. After washing with tap water for 5 min, the slides were dehydrated and mounted using synthetic mounting solution. Stained sections were visualized with a fluorescent microscope (EVOS FL Auto 2, Thermo Fisher), and staining intensity was measured with ImageJ software (NIH).

### Chromatin immunoprecipitation (ChIP) assay

Chromatin fragmentation was performed by sonication in ChIP lysis buffer (50 mM Tris-HCl (pH 8.1), 1% SDS, 10 mM EDTA (pH 7.6), and freshly added protease inhibitor cocktail). Chromatin extracts containing DNA fragments with an average of 250 bp were then diluted 10 times with dilution buffer (1% Triton X-100, 2  mM EDTA, 150 mM NaCl, 20 mM Tris-HCl (pH 8.1), and freshly added protease inhibitor cocktail) and subjected to immunoprecipitations overnight at 4°C. Immunocomplexes were captured by incubating 40 μl of protein A/G sepharose for 2 h at 4°C. Beads were washed with TSE I buffer (0.1% SDS, 1% Triton X-100, 2 mM EDTA, 20 mM Tris-HCl (pH 8.1), and 150 mM NaCl), TSE II buffer (0.1% SDS, 1% Triton X-100, 2 mM EDTA, 20 mM Tris-HCl (pH 8.1), and 500 mM NaCl), buffer III (0.25 M LiCl, 1% NP-40, 1% deoxycholate, 10 mM Tris-HCl (pH 8.1), and 1 mM EDTA), TE buffer (10 mM Tris-HCl (pH 8.0) and 1 mM EDTA) for 3 times, and eluted in elution buffer (1% SDS and 0.1 M NaHCO_3_). Crosslinking was reversed overnight at 65°C in elution buffer, and DNA was purified with a QIAquick Gel Extraction Kit (QIAGEN). Precipitated DNA was analyzed by quantitative RT-PCR. For quantitative real-time PCR analysis, 2 μl from 50 μl DNA extractions was used. For Re-ChIP assay, second round of ChIP was performed using the elution of the first round ChIP. The result was calculated relative to the original input using the same amount of DNA in the qPCRs. Primer sequences are appended in S5 Table.

### Co-Immunoprecipitation (Co-IP)

C2C12 cell line was used to Co-IP experiment. Transfection of each indicated gene was performed using jetPRIME transfection reagent (Polyplus) following the protocol provided from the manufacturer. Lysis of cell was performed by sonication in Co-IP lysis buffer (Pierce, Thermo Scientific) mixed with 1× protease inhibitor (Halt protease inhibitor cocktail, Thermo Scientific). Lysates were subjected to precipitations with Ni-NTA agarose bead (QIAGEN) for overnight at 4°C. Beads were washed with Ni-NTA wash buffer (20 mM Tris, 150 mM NaCl, imidazole 20 mM (pH 8.0)) for 6 times. Elution of precipitated protein was performed by adding 1× Laemmli sample buffer to bead and boiled in 95°C for 10 min. Eluted sample was used for following western blot analysis.

### Blood lactate level measurement

The concentration of blood lactate was measured before the onset of running and immediately after exhaustion by treadmill running. Exhaustion was determined by the inability of the mice to run on the treadmill more than 10 s despite stimulation. The measurement was performed with lactate meter (Lactate pro 2, LT-1730, ARKRAY) using blood from a tail tip.

### Targeted metabolomics analysis

Targeted metabolomics analysis was performed as previously described [106–110]. Briefly, metabolites were extracted from freshly dissected Q muscle at the exhaustion of the treadmill running. Dissected Q muscle was frozen in liquid nitrogen and ground into powder. Approximately 20 mg of muscle powder was mixed with 500 μl of LC-MS grade methanol (Merck) and 10 mM of the external standard. The mixture was sonicated with 3 cycles of 40 s on-state and 30 s off-state (Bioruptor Pico, Diagenode). After adding 500 μl of LC-MS grade chloroform (Merck) and 200 μl of LC-MS grade water (Merck), the mixture and incubated for 10 min at 4°C. Following the centrifugation with 13,000 rpm for 10 min at 4°C, the upper phase was collected in a 5-mL tube, was frozen using liquid nitrogen for 15 min, and was lyophilized for 48 h (Lyophilizer, FD8508, ilShinBioBase). The lyophilized product was dissolved with the solvent and was subjected to LC-MS/MS.

### ATP level measurement

ATP assay kit (ATP bioluminescence assay kit HS II, Roche) was used for ATP analysis, and all experimental procedures were performed according to given protocol from manufacturer. Lysates were extracted from 10 mg of freshly isolated quadriceps after 100 min of treadmill run. Luminescence signal was detected by luminometer (LB 96V microplate luminometer, Berthold) and normalized to protein concentration of lysates (M/g protein).

### Statistical analysis

Data are presented as mean ± SEM, a column showing the mean and an error bar showing the SEM. Statistical analyses were performed using GraphPad Prism (GraphPadSoftware) and were indicated in figure legends. *p*-Values of less 0.05 were considered statistically significant.

## Supporting information

S1 Fig Baf155 is dispensable for the development and maturation of skeletal muscle. **(A)** Body weight of age and sex matched Baf155^WT^ and Baf155^ΔMF^ mice (*n* = 20 mice per each genotype). **(B)** Representative appearance of hind limb skeletal muscles (TA, EDL, GA, SOL, Q) of Baf155^WT^ and Baf155^ΔMF^ mice. Scale bar, 1 cm. **(C)** Muscle weight normalized to body weight of hind limb skeletal muscles of Baf155^WT^ and Baf155^ΔMF^ mice (*n* = 3 mice per each genotype). **(D)** Total body DEXA analysis of Baf155^WT^ and Baf155^ΔMF^ mice (*n* = 15 mice per each genotype). **(E)** Representative HE staining of TA muscles of Baf155^WT^ (upper panel) and Baf155 ^ΔMF^ mice (lower panel). Scale bars, 100 μm for left column and 200 μm for right column. **(F)** Representative IHC staining image of Pax7, DAPI, and Laminin in TA muscle of Baf155^WT^ (upper panel) and Baf155^ΔMF^ mice (lower panel). Scale bars, 100 μm. **(G)** Quantification of the myofiber number of per cross section in TA muscle of Baf155^WT^ and Baf155^ΔMF^ mice. **(H)** Frequency of myofibers within each indicated CSA range in TA of Baf155^WT^ and Baf155^ΔMF^ mice. **(I)** The number of Pax7^+^ cell per 100 MFs in TA of Baf155^WT^ and Baf155^ΔMF^ mice. Three biological replicates of (F) were performed and quantified for (G–I) (*n* = 3 mice per each genotype). Each dot in the graphs (A, C, D, G–I) represents each mouse (biological replicate). Data are presented as mean ± SEM of biological replicates. Statistical analyses were performed using unpaired Student’s *t* test (n.s., not significant versus control) (A, C, D, G, and I). The data underlying this figure can be found in S1 Data. Baf155, Brg1/Brm-associated factor 155; CSA, cross-sectional area; DEXA, dual-energy X-ray absorptiometry; EDL, extensor digitorum longus; GA, gastrocnemius; HE, hematoxylin and eosin; IHC, immunohistochemistry; MF, myofiber; Pax7, paired box 7; Q, quadriceps; SEM, standard error of the mean; SOL, soleus; TA, tibialis anterior.
(TIF)

S2 Fig Baf155 ablation in cardiac muscle does not affect exercise capacity. **(A)** RT-qPCR analysis of *Baf155* in each indicated organ from Baf155^WT^ and Baf155^ΔMF^ mice (*n* = 3 mice per each genotype). **(B)** Representative immunoblotting analyses of Baf155 in each indicated organ from Baf155^WT^ and Baf155^ΔMF^ mice. Each lane in the immunoblotting image indicates each mouse. **(C)** The densitometric quantification of relative protein level of Baf155 in each indicated organ of Baf155^ΔMF^ mice compared to Baf155^WT^ mice (*n* = 3 mice per each genotype). **(D)** Schematic representation of the experimental strategies of tamoxifen treatment and treadmill running test. To ablate Baf155 in cardiac muscle, tamoxifen (20 mg/mL in corn oil) was administered orally to Baf155^WT^ or Baf155^ΔCMF^ mice for 5 consecutive days (160 mg/kg body weight/day). To measure endurance exercise capacity, mice were subjected to treadmill running following 3 days of acclimation at the age of 3 months. **(E)** RT-qPCR analysis of *Baf155* in cardiac and Q muscles from Baf155^WT^ and Baf155^ΔCMF^ mice after tamoxifen treatment (*n* = 4 mice per each genotype). **(F)** Representative immunoblotting analyses of Baf155 in each indicated muscle from Baf155^WT^ and Baf155^ΔCMF^ mice. Each lane in the immunoblotting image indicates each mouse. **(G)** The densitometric quantification of relative protein level of Baf155 in each indicated muscle of Baf155^ΔCMF^ mice compared to Baf155^WT^ mice (*n* = 3 mice per each genotype). **(H)** Weight of cardiac muscle of Baf155^WT^ and Baf155^ΔCMF^ mice after tamoxifen treatment (*n* = 6 mice per each genotype). **(I, J)** The measurement values of treadmill running test. Total running time (min) (I) and total running distance (m) (J) (*n* = 7 mice per genotype). Each dot in the graphs (A, C, E, G–J) represents each mouse (biological replicate). Data are presented as mean ± SEM of biological replicates. Statistical analyses were performed using unpaired Student’s *t* test (n.s., not significant; *P < 0.05; **P < 0.01; ***P < 0.001 versus Baf155^WT^ control). The data underlying this figure can be found in S1 Data. Baf155, Brg1/Brm-associated factor 155; CMF, cardiac myofiber; kg, kilogram; MF, myofiber; mg, milligram; mL, milliliter; Q, quadriceps; RT-qPCR, reverse transcription quantitative real-time PCR; SEM, standard error of the mean.
(TIF)

S3 Fig Fiber type composition or mitochondrial function are comparable between Baf155^WT^ and Baf155^ΔMF^ mice. **(A)** Representative IHC staining image of MyHC1, MyHC2a, and MyHC2b in TA muscle of Baf155^WT^ (upper panel) and Baf155^ΔMF^ mice (lower panel). Scale bars, 100 μm. **(B)** Quantification of the number of each indicated fiber type in TA muscle of Baf155^WT^ and Baf155^ΔMF^ mice. Three or 4 biological replicates of (A) were performed and quantified (*n* = 3–4 mice per each genotype). **(C)** RT-qPCR analysis of each indicated fiber type in TA muscle of Baf155^WT^ and Baf155^ΔMF^ mice (*n* = 3–4 mice per each genotype). **(D)** Representative NADH staining image in TA muscle of Baf155^WT^ and Baf155^ΔMF^ mice. Scale bars, 200 μm. **(E)** Quantification of the number of NADH positive myofiber (E) and quantification of the NADH staining intensity in TA muscle of Baf155^WT^ and Baf155^ΔMF^ mice. **(F)** The quantification of relative staining intensity of whole section. Three biological replicates of (D) were performed and quantified for (E and F) (*n* = 3 mice per each genotype). **(G, H)** RT-qPCR analysis of nuclear encoded (G) and mitochondrial encoded genes (H), which are related to mitochondrial function, in TA muscle of Baf155^WT^ and Baf155^ΔMF^ mice (*n* = 3 mice per each genotype). Each dot in the graphs (B, C, E–H) represents each mouse (biological replicate). Data are presented as mean ± SEM of biological replicates. Statistical analyses were performed using unpaired Student’s *t* test (n.s., not significant; ***P < 0.001 versus Baf155^WT^ control). The data underlying this figure can be found in S1 Data. Baf155, Brg1/Brm-associated factor 155; IHC, immunohistochemistry; MF, myofiber; MyHC, myosin heavy chain; NADH, nicotinamide adenine dinucleotide hydrogen; RT-qPCR, reverse transcription quantitative real-time PCR; SEM, standard error of the mean; TA, tibialis anterior.
(TIF)

S4 Fig Baf155^ΔMF^ mice are not susceptible to the metabolic defect in response to high-fat feeding. **(A)** Body weight after high-fat feeding and **(B)** percentage of weight gain compared to body weight before high-fat feeding. **(C–E)** The value of mass normalized to body weight; limb muscle (C), fat (D), and liver (E). **(F–I)** Glucose tolerance test. Blood glucose level of Baf155WT and Baf155 ^ΔMF^ mice with normal chow (F) and area under curve of blood glucose level with normal chow (G). Blood glucose level of Baf155^WT^ and Baf155 ^ΔMF^ mice after high-fat feeding (H) and area under curve of blood glucose level after high-fat feeding (I). Each dot in the graphs (A–E, G, and I) represents each mouse (biological replicate). Data are presented as mean ± SEM of biological replicates. Statistical analyses were performed using unpaired Student’s *t* test (n.s., not significant). The data underlying this figure can be found in S1 Data. Baf155, Brg1/Brm-associated factor 155; SEM, standard error of the mean.
(TIF)

S5 Fig Schematic model of the role of Baf155 in energy metabolism regulation through HIF-1α signaling in skeletal muscle. Baf155 mediates DNA binding of HIF-1α. This regulatory role requires DNA binding of STAT3, which forms a coactivator complex with HIF-1α. Baf155 ablation attenuates HIF-1α signaling, which leads to the alteration of energy metabolism, in skeletal muscle and enhances endurance exercise capacity. Baf155, Brg1/Brm-associated factor 155; HIF-1α, hypoxia inducible factor-1α; STAT3, signal transducer and activator of transcription 3.
(TIF)

S1 Table Annotated genes in GO term analysis. Genes of DEGs annotated in each indicated biological process according to GO term analysis. ADAMTS, a disintegrin-like metalloproteinase with thrombospondin motif type1; CCL, chemokine (C-C motif) ligand; CISH, cytokine inducible SH2 containing protein; DEG, differentially expressed gene; HAS, hyaluronan synthase; MNDAL, myeloid cell nuclear differentiation antigen like; NOS, nitricoxide synthase; PF, Platelet factor; SMPD, sphingomyelin phosphodiesterase; SOCS, suppressor of cytokine signaling.
(TIFF)

S2 Table Annotated genes in KEGG pathway analysis. Genes of DEGs annotated in each indicated biological process according to the KEGG pathway analysis. ADAMTS, a disintegrin-like metalloproteinase with thrombospondin motif type1; CCL, chemokine (C-C motif) ligand; CISH, cytokine inducible SH2 containing protein; DEG, differentially expressed gene; PF, platelet factor; SMPD, sphingomyelin phosphodiesterase; SOCS, suppressor of cytokine signaling.
(TIFF)

S3 Table Annotated genes in IPA. Genes of DEGs annotated in each indicated signaling pathway according to the IPA. CISH, cytokine inducible SH2 containing protein; DEG, differentially expressed gene; SOCS, suppressor of cytokine signaling.
(TIFF)

S4 Table RT-qPCR primer sequences. The primer sequences for RT-qPCR targeting each indicated gene. Baf155, Brg1/Brm-associated factor 155; CISH, cytokine inducible SH2 containing protein; F, forward; Glut, glucose transporter; LDHA, lactate dehydrogenase A; MyHC, myosin heavy chain; Pgk, phosphoglycerate kinase; R, reverse; RT-qPCR, reverse transcription-quantitative polymerase chain reaction; SOCS, suppressor of cytokine signaling.
(TIFF)

S5 Table ChIP qPCR primers. The primer sequences for ChIP qPCR targeting each indicated promoter of gene. CISH, cytokine inducible SH2 containing protein; ChIP-qPCR, chromatin immunoprecipitation-quantitative polymerase chain reaction; F, forward; Glut, glucose transporter; HIF, hypoxia inducible factor; LDHA, lactate dehydrogenase A; Pgk, phosphoglycerate kinase; R, reverse; SOCS, suppressor of cytokine signaling.
(TIFF)

S6 Table False positive risks (FPRs) of data with one-star *p*-value. The FPRs of the data with one-star *p*-value (0.01<*p*<0.05). Information of data was presented as the number of figure-panel label and the purpose of the experiment.
(TIF)

S1 Data Excel spreadsheet containing the underlying numerical data for Figs 1A, 1C, 1E, 1F, 1H, 2B, 2C, 2D, 2E, 2F, 3A, 3B, 3C, 3D, 3F, 4B, 4C, 4F, 4G, 5E, 6C, 6D, 6E, 6F, 6G, 7B, 7D, 8A, 8B, 8C, 8D, 8F, 8H, 8J, 8K, S1A, S1C, S1D, S1G, S1H, S1I, S2A, S2C, S2E, S2G, S2H, S2I, S2J, S3B, S3C, S3E, S3F, S3G, S3H, S4A, S4B, S4C, S4D, S4E, S4F, S4G, S4H, and S4I. (XLSX)

S1 Raw Image Uncropped raw images of western blot data. The red square within each image indicates the cropped area for the representative image.
(PDF)

## Abbreviations

AMPK: adenosine monophosphate-activated protein kinase
ATP: adenosine triphosphate
CCD: coiled-coil domain
ChIP: chromatin immunoprecipitation
CSA: cross-sectional area
DEG: differentially expressed gene
DEXA: dual-energy X-ray absorptiometry
FPR: false positive risk
GEO: Gene Expression Omnibus
GO: Gene Ontology
GTT: glucose tolerance test
HIF: hypoxia-inducible factor
HIF-1α: hypoxia-inducible factor-1α
HRE: hypoxia response element
IHC: immunohistochemistry
IPA: ingenuity pathway analysis
KEGG: Kyoto Encyclopedia of Genes and Genomes
MyHC: myosin heavy chain
NADH: nicotinamide adenine dinucleotide hydrogen
OCT: optimal cutting temperature
RT: room temperature
RT-qPCR: reverse transcription-quantitative PCR
SOCS: suppressors of cytokine signaling
SRE: STAT3 response element
SWI/SNF: switch/sucrose non-fermentable
