# Supplementary figures and images for: Baf155 regulates skeletal muscle metabolism via HIF-1a signaling
Source: PLoS Biol. 2023 Jul 21;21(7):e3002192. doi: 10.1371/journal.pbio.3002192 (PMC10396025; doi:10.1371/journal.pbio.3002192)

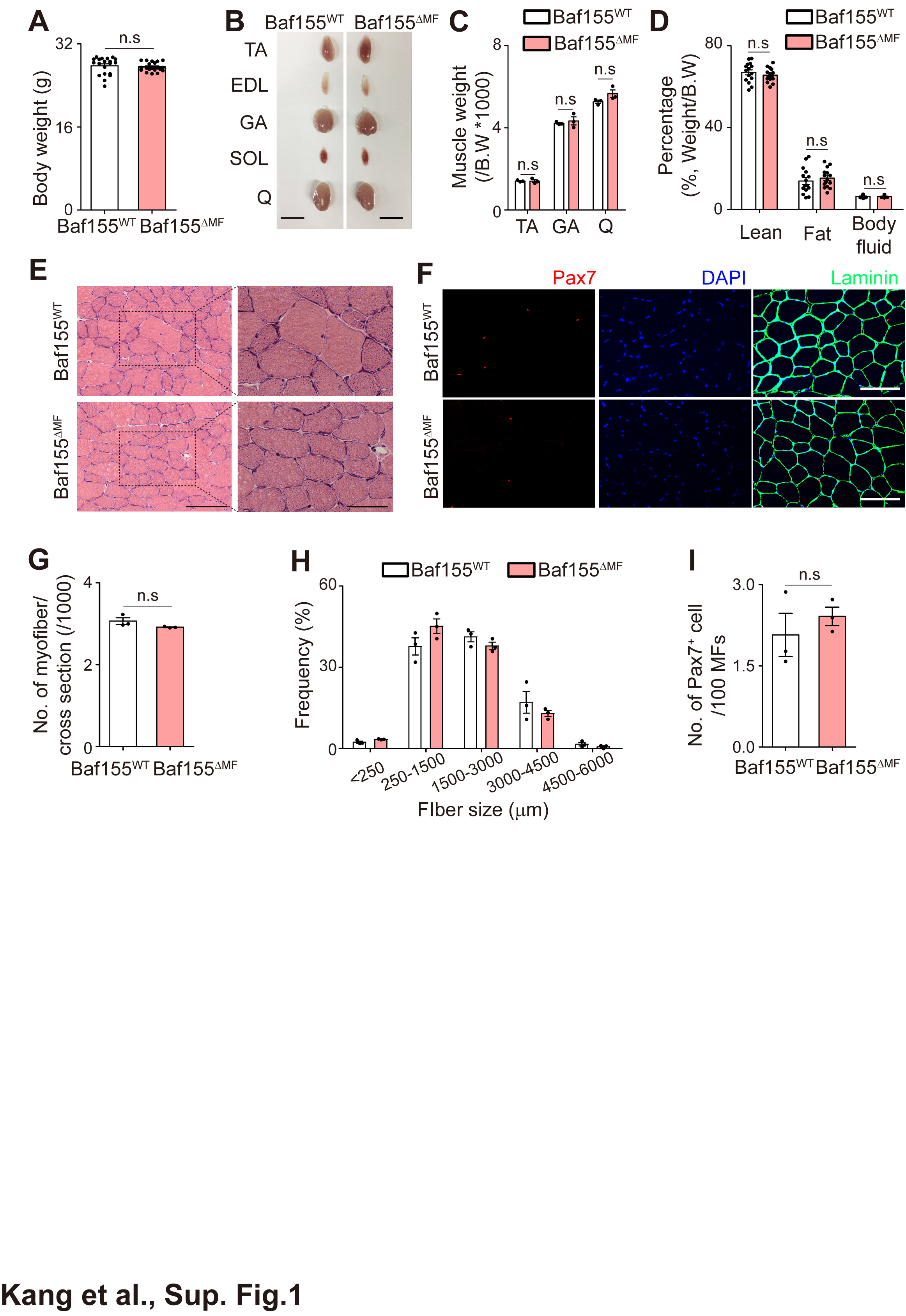

Supplement: S1 Fig — (A) Body weight of age and sex matched Baf155WT and Baf155ΔMF mice (n = 20 mice per each genotype). (B) Representative appearance of hind limb skeletal muscles (TA, EDL, GA, SOL, Q) of Baf155WT and Baf155ΔMF mice. Scale bar, 1 cm. (C) Muscle weight normalized to body weight of hind limb skeletal muscles of Baf155WT and Baf155ΔMF mice (n = 3 mice per each genotype). (D) Total body DEXA analysis of Baf155WT and Baf155ΔMF mice (n = 15 mice per each genotype). (E) Representative HE staining of TA muscles of Baf155WT (upper panel) and Baf155 ΔMF mice (lower panel). Scale bars, 100 μm for left column and 200 μm for right column. (F) Representative IHC staining image of Pax7, DAPI, and Laminin in TA muscle of Baf155WT (upper panel) and Baf155ΔMF mice (lower panel). Scale bars, 100 μm. (G) Quantification of the myofiber number of per cross section in TA muscle of Baf155WT and Baf155ΔMF mice. (H) Frequency of myofibers within each indicated CSA range in TA of Baf155WT and Baf155ΔMF mice. (I) The number of Pax7+ cell per 100 MFs in TA of Baf155WT and Baf155ΔMF mice. Three biological replicates of (F) were performed and quantified for (G–I) (n = 3 mice per each genotype). Each dot in the graphs (A, C, D, G–I) represents each mouse (biological replicate). Data are presented as mean ± SEM of biological replicates. Statistical analyses were performed using unpaired Student’s t test (n.s., not significant versus control) (A, C, D, G, and I). The data underlying this figure can be found in S1 Data. Baf155, Brg1/Brm-associated factor 155; CSA, cross-sectional area; DEXA, dual-energy X-ray absorptiometry; EDL, extensor digitorum longus; GA, gastrocnemius; HE, hematoxylin and eosin; IHC, immunohistochemistry; MF, myofiber; Pax7, paired box 7; Q, quadriceps; SEM, standard error of the mean; SOL, soleus; TA, tibialis anterior. (TIF) [file pbio.3002192.s001.tif]

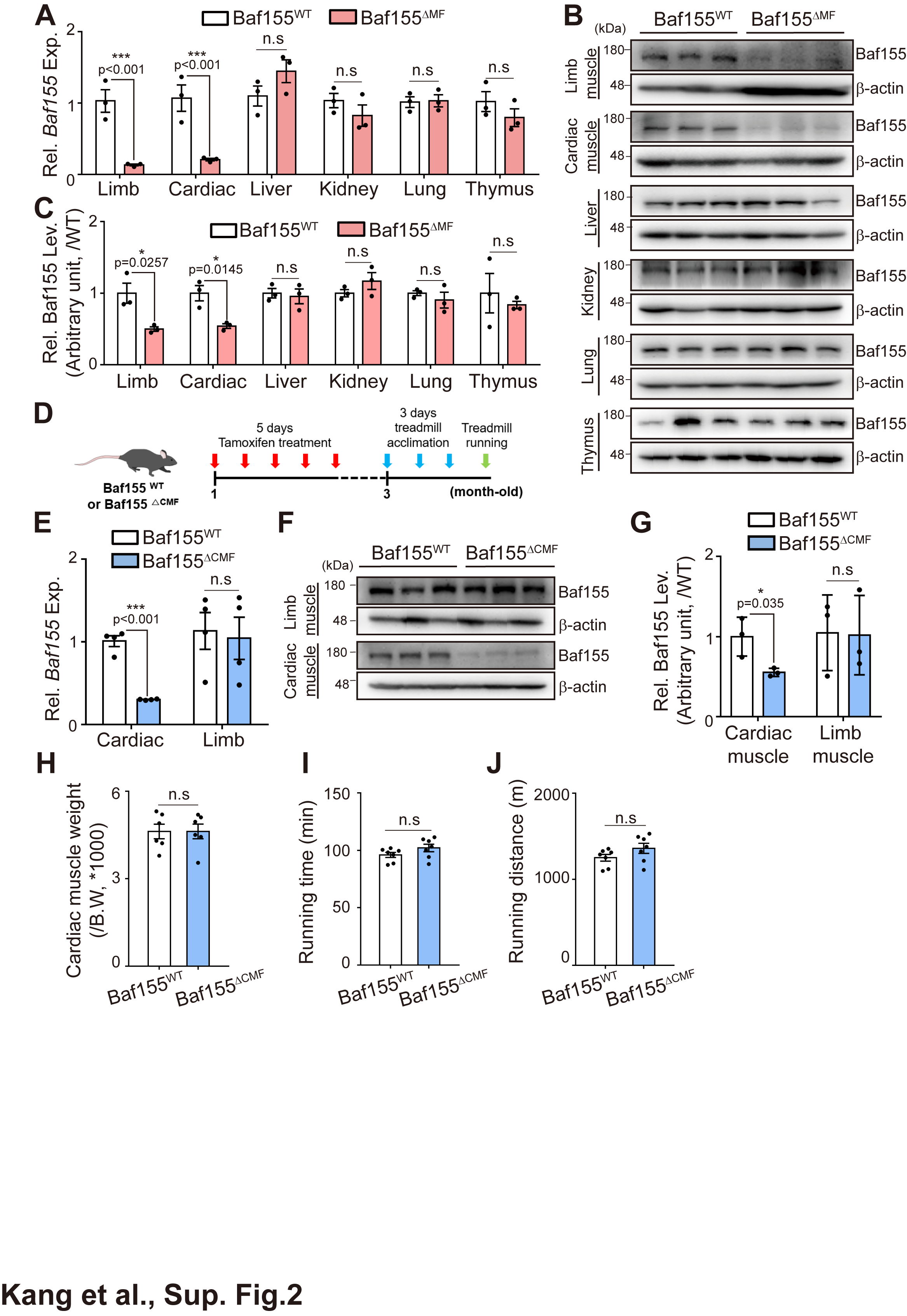

Supplement: S2 Fig — (A) RT-qPCR analysis of Baf155 in each indicated organ from Baf155WT and Baf155ΔMF mice (n = 3 mice per each genotype). (B) Representative immunoblotting analyses of Baf155 in each indicated organ from Baf155WT and Baf155ΔMF mice. Each lane in the immunoblotting image indicates each mouse. (C) The densitometric quantification of relative protein level of Baf155 in each indicated organ of Baf155ΔMF mice compared to Baf155WT mice (n = 3 mice per each genotype). (D) Schematic representation of the experimental strategies of tamoxifen treatment and treadmill running test. To ablate Baf155 in cardiac muscle, tamoxifen (20 mg/mL in corn oil) was administered orally to Baf155WT or Baf155ΔCMF mice for 5 consecutive days (160 mg/kg body weight/day). To measure endurance exercise capacity, mice were subjected to treadmill running following 3 days of acclimation at the age of 3 months. (E) RT-qPCR analysis of Baf155 in cardiac and Q muscles from Baf155WT and Baf155ΔCMF mice after tamoxifen treatment (n = 4 mice per each genotype). (F) Representative immunoblotting analyses of Baf155 in each indicated muscle from Baf155WT and Baf155ΔCMF mice. Each lane in the immunoblotting image indicates each mouse. (G) The densitometric quantification of relative protein level of Baf155 in each indicated muscle of Baf155ΔCMF mice compared to Baf155WT mice (n = 3 mice per each genotype). (H) Weight of cardiac muscle of Baf155WT and Baf155ΔCMF mice after tamoxifen treatment (n = 6 mice per each genotype). (I, J) The measurement values of treadmill running test. Total running time (min) (I) and total running distance (m) (J) (n = 7 mice per genotype). Each dot in the graphs (A, C, E, G–J) represents each mouse (biological replicate). Data are presented as mean ± SEM of biological replicates. Statistical analyses were performed using unpaired Student’s t test (n.s., not significant; *P < 0.05; **P < 0.01; ***P < 0.001 versus Baf155WT control). The data underlying this figure can be found in S1 D [file pbio.3002192.s002.tif]

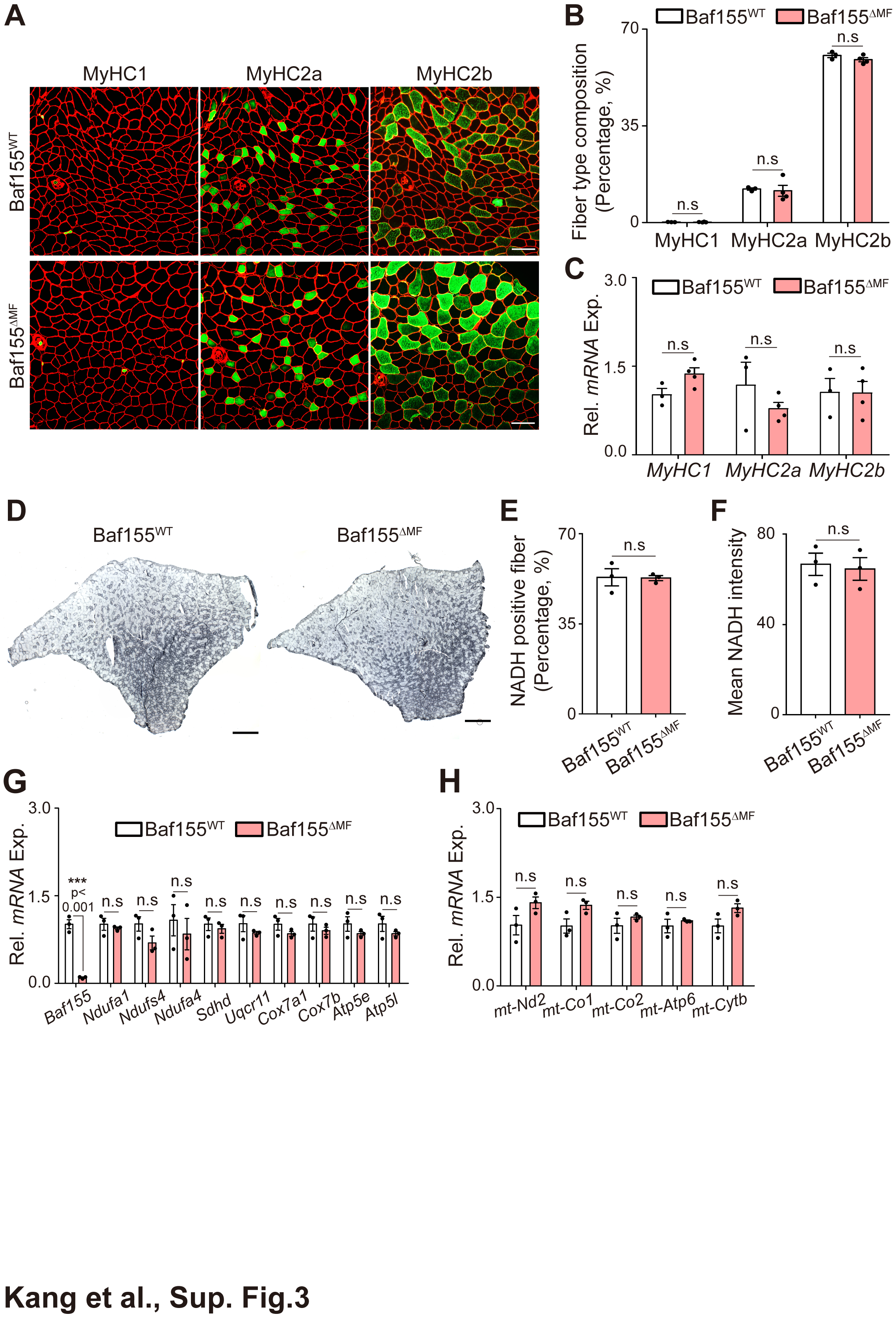

Supplement: S3 Fig — (A) Representative IHC staining image of MyHC1, MyHC2a, and MyHC2b in TA muscle of Baf155WT (upper panel) and Baf155ΔMF mice (lower panel). Scale bars, 100 μm. (B) Quantification of the number of each indicated fiber type in TA muscle of Baf155WT and Baf155ΔMF mice. Three or 4 biological replicates of (A) were performed and quantified (n = 3–4 mice per each genotype). (C) RT-qPCR analysis of each indicated fiber type in TA muscle of Baf155WT and Baf155ΔMF mice (n = 3–4 mice per each genotype). (D) Representative NADH staining image in TA muscle of Baf155WT and Baf155ΔMF mice. Scale bars, 200 μm. (E) Quantification of the number of NADH positive myofiber (E) and quantification of the NADH staining intensity in TA muscle of Baf155WT and Baf155ΔMF mice. (F) The quantification of relative staining intensity of whole section. Three biological replicates of (D) were performed and quantified for (E and F) (n = 3 mice per each genotype). (G, H) RT-qPCR analysis of nuclear encoded (G) and mitochondrial encoded genes (H), which are related to mitochondrial function, in TA muscle of Baf155WT and Baf155ΔMF mice (n = 3 mice per each genotype). Each dot in the graphs (B, C, E–H) represents each mouse (biological replicate). Data are presented as mean ± SEM of biological replicates. Statistical analyses were performed using unpaired Student’s t test (n.s., not significant; ***P < 0.001 versus Baf155WT control). The data underlying this figure can be found in S1 Data. Baf155, Brg1/Brm-associated factor 155; IHC, immunohistochemistry; MF, myofiber; MyHC, myosin heavy chain; NADH, nicotinamide adenine dinucleotide hydrogen; RT-qPCR, reverse transcription quantitative real-time PCR; SEM, standard error of the mean; TA, tibialis anterior. (TIF) [file pbio.3002192.s003.tif]

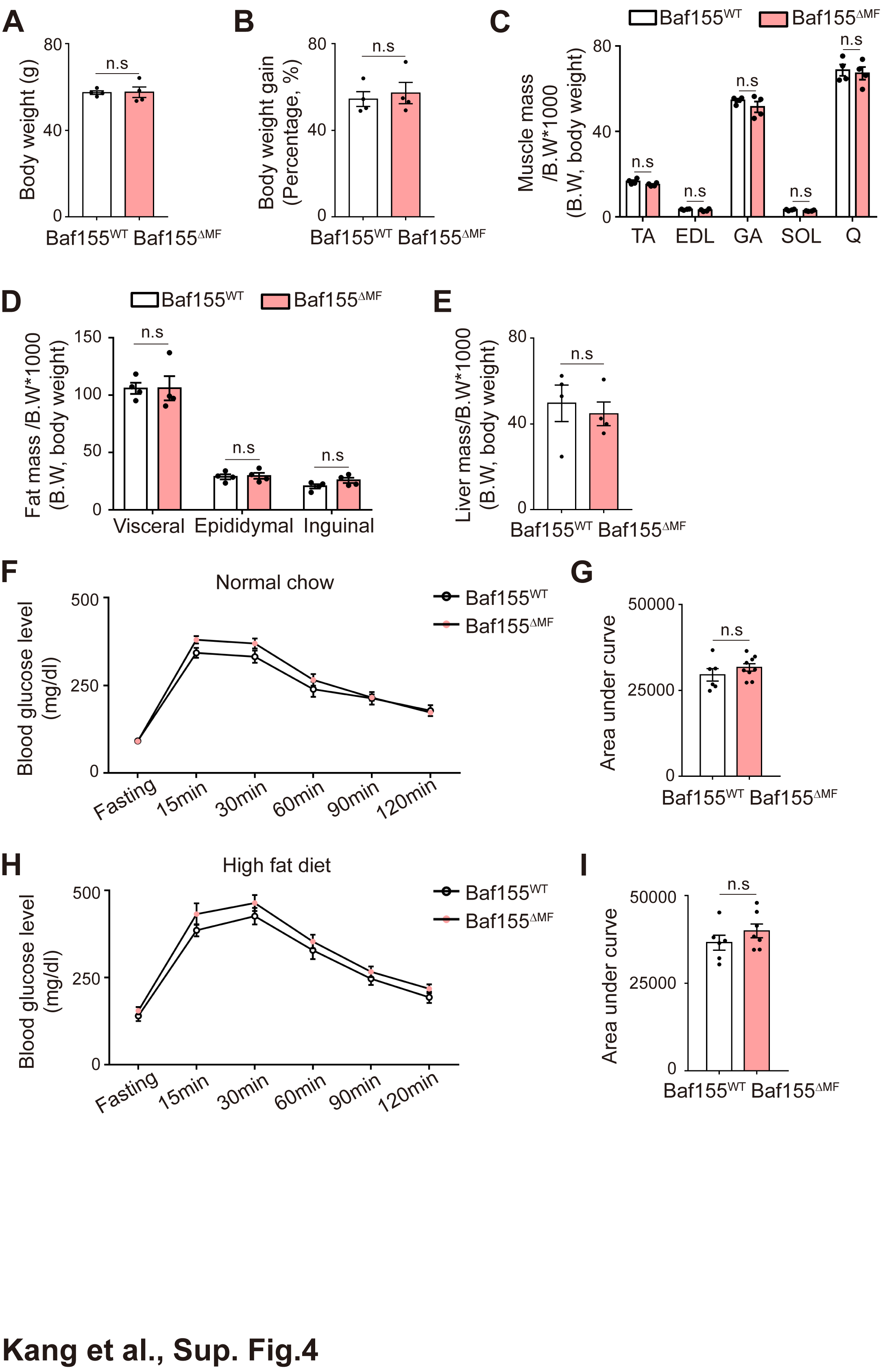

Supplement: S4 Fig — (A) Body weight after high-fat feeding and (B) percentage of weight gain compared to body weight before high-fat feeding. (C–E) The value of mass normalized to body weight; limb muscle (C), fat (D), and liver (E). (F–I) Glucose tolerance test. Blood glucose level of Baf155WT and Baf155 ΔMF mice with normal chow (F) and area under curve of blood glucose level with normal chow (G). Blood glucose level of Baf155WT and Baf155 ΔMF mice after high-fat feeding (H) and area under curve of blood glucose level after high-fat feeding (I). Each dot in the graphs (A–E, G, and I) represents each mouse (biological replicate). Data are presented as mean ± SEM of biological replicates. Statistical analyses were performed using unpaired Student’s t test (n.s., not significant). The data underlying this figure can be found in S1 Data. Baf155, Brg1/Brm-associated factor 155; SEM, standard error of the mean. (TIF) [file pbio.3002192.s004.tif]

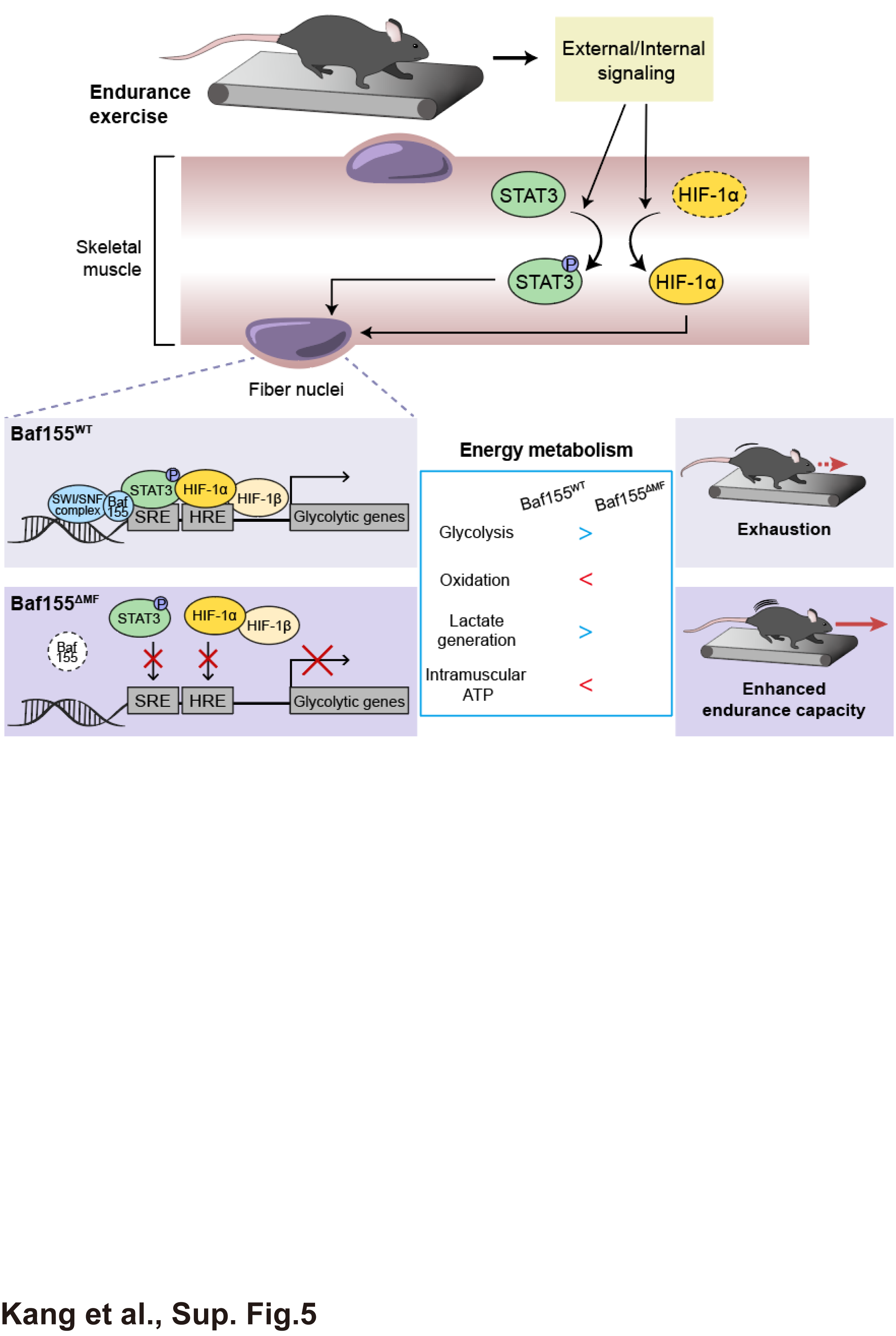

Supplement: S5 Fig — Baf155 mediates DNA binding of HIF-1α. This regulatory role requires DNA binding of STAT3, which forms a coactivator complex with HIF-1α. Baf155 ablation attenuates HIF-1α signaling, which leads to the alteration of energy metabolism, in skeletal muscle and enhances endurance exercise capacity. Baf155, Brg1/Brm-associated factor 155; HIF-1α, hypoxia inducible factor-1α; STAT3, signal transducer and activator of transcription 3. (TIF) [file pbio.3002192.s005.tif]

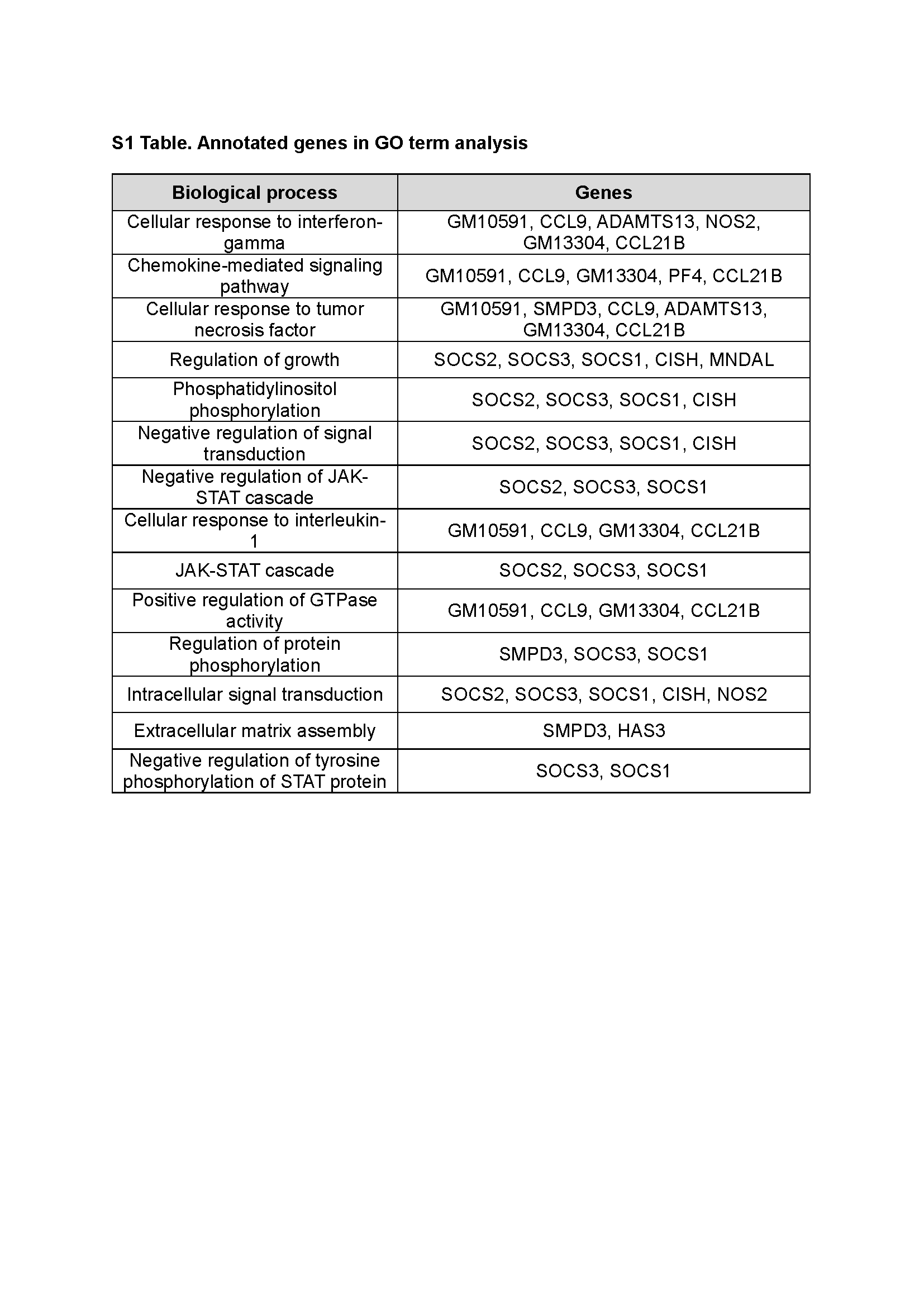

Supplement: S1 Table — Genes of DEGs annotated in each indicated biological process according to GO term analysis. ADAMTS, a disintegrin-like metalloproteinase with thrombospondin motif type1; CCL, chemokine (C-C motif) ligand; CISH, cytokine inducible SH2 containing protein; DEG, differentially expressed gene; HAS, hyaluronan synthase; MNDAL, myeloid cell nuclear differentiation antigen like; NOS, nitricoxide synthase; PF, Platelet factor; SMPD, sphingomyelin phosphodiesterase; SOCS, suppressor of cytokine signaling. (TIFF) [file pbio.3002192.s006.tiff]

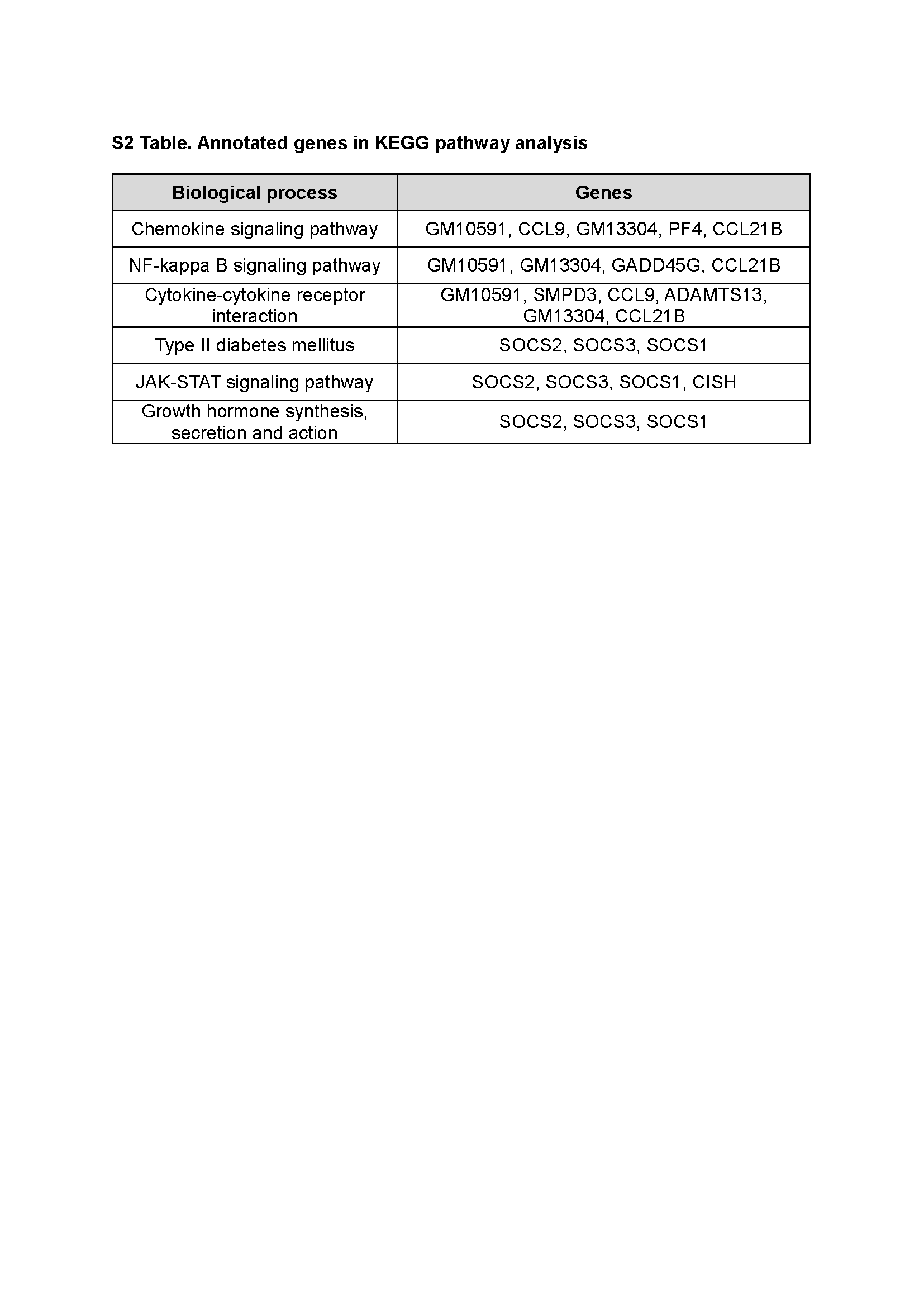

Supplement: S2 Table — Genes of DEGs annotated in each indicated biological process according to the KEGG pathway analysis. ADAMTS, a disintegrin-like metalloproteinase with thrombospondin motif type1; CCL, chemokine (C-C motif) ligand; CISH, cytokine inducible SH2 containing protein; DEG, differentially expressed gene; PF, platelet factor; SMPD, sphingomyelin phosphodiesterase; SOCS, suppressor of cytokine signaling. (TIFF) [file pbio.3002192.s007.tiff]

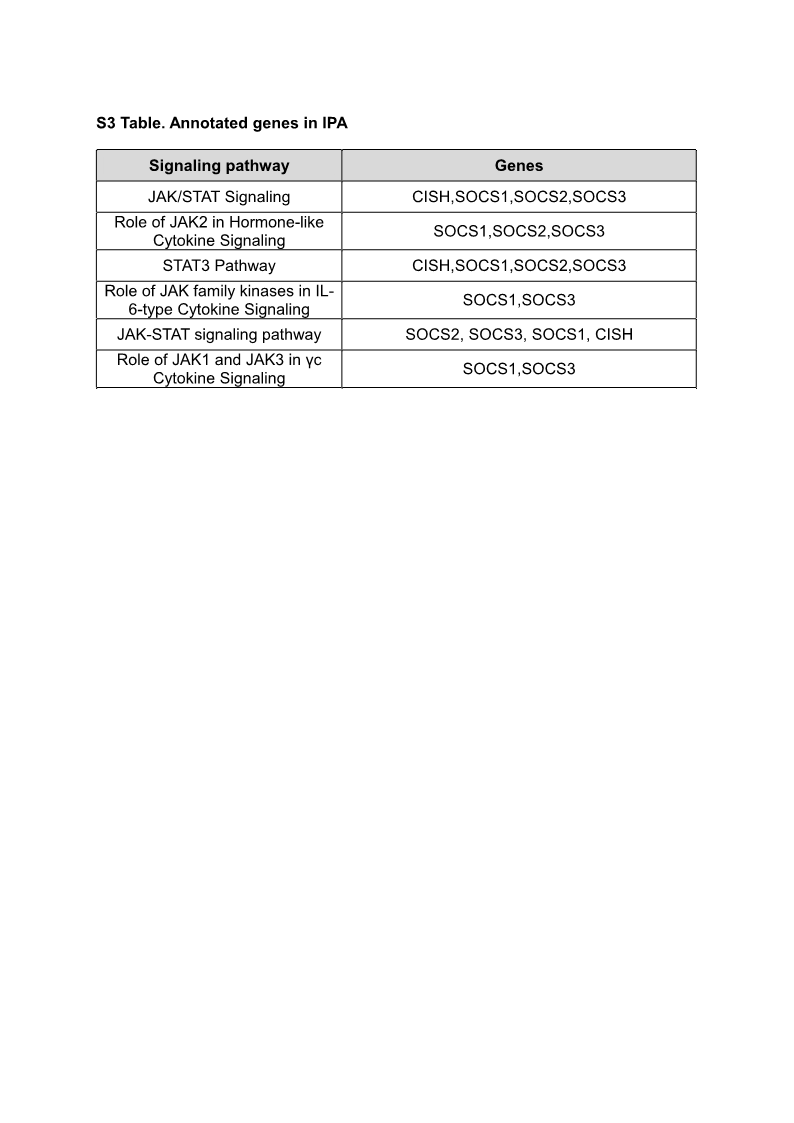

Supplement: S3 Table — Genes of DEGs annotated in each indicated signaling pathway according to the IPA. CISH, cytokine inducible SH2 containing protein; DEG, differentially expressed gene; SOCS, suppressor of cytokine signaling. (TIFF) [file pbio.3002192.s008.tiff]

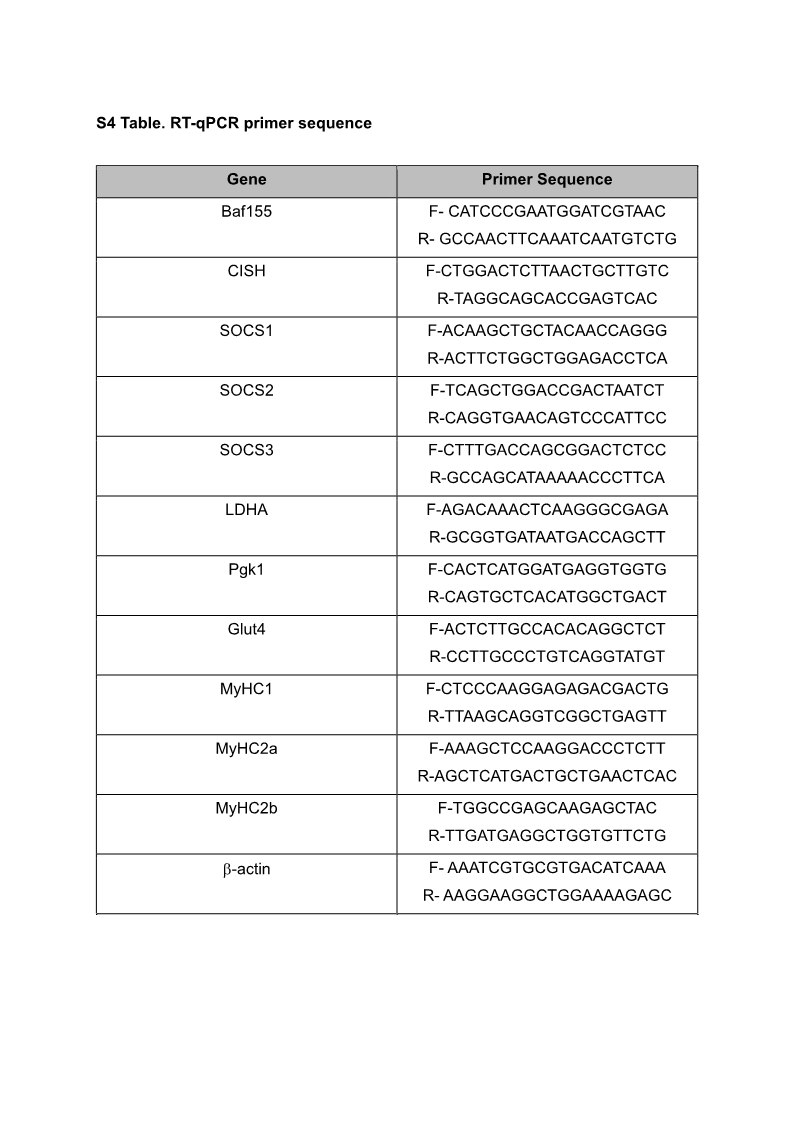

Supplement: S4 Table — The primer sequences for RT-qPCR targeting each indicated gene. Baf155, Brg1/Brm-associated factor 155; CISH, cytokine inducible SH2 containing protein; F, forward; Glut, glucose transporter; LDHA, lactate dehydrogenase A; MyHC, myosin heavy chain; Pgk, phosphoglycerate kinase; R, reverse; RT-qPCR, reverse transcription-quantitative polymerase chain reaction; SOCS, suppressor of cytokine signaling. (TIFF) [file pbio.3002192.s009.tiff]

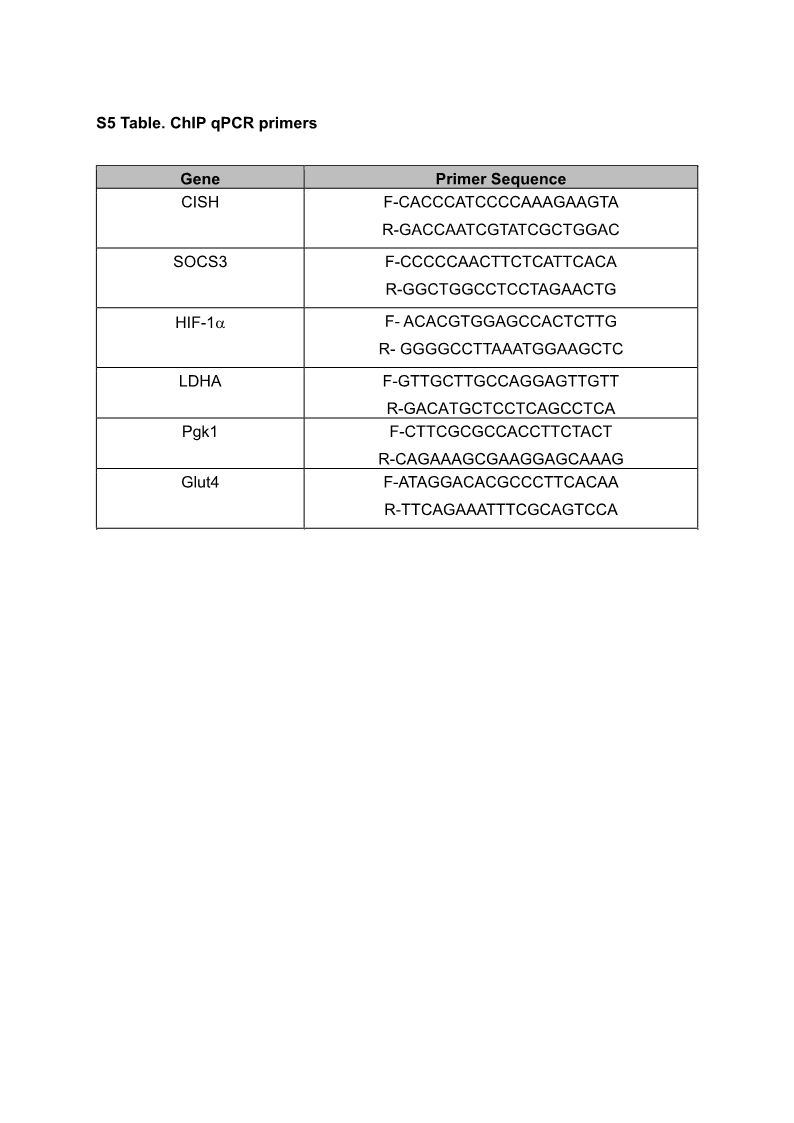

Supplement: S5 Table — The primer sequences for ChIP qPCR targeting each indicated promoter of gene. CISH, cytokine inducible SH2 containing protein; ChIP-qPCR, chromatin immunoprecipitation-quantitative polymerase chain reaction; F, forward; Glut, glucose transporter; HIF, hypoxia inducible factor; LDHA, lactate dehydrogenase A; Pgk, phosphoglycerate kinase; R, reverse; SOCS, suppressor of cytokine signaling. (TIFF) [file pbio.3002192.s010.tiff]

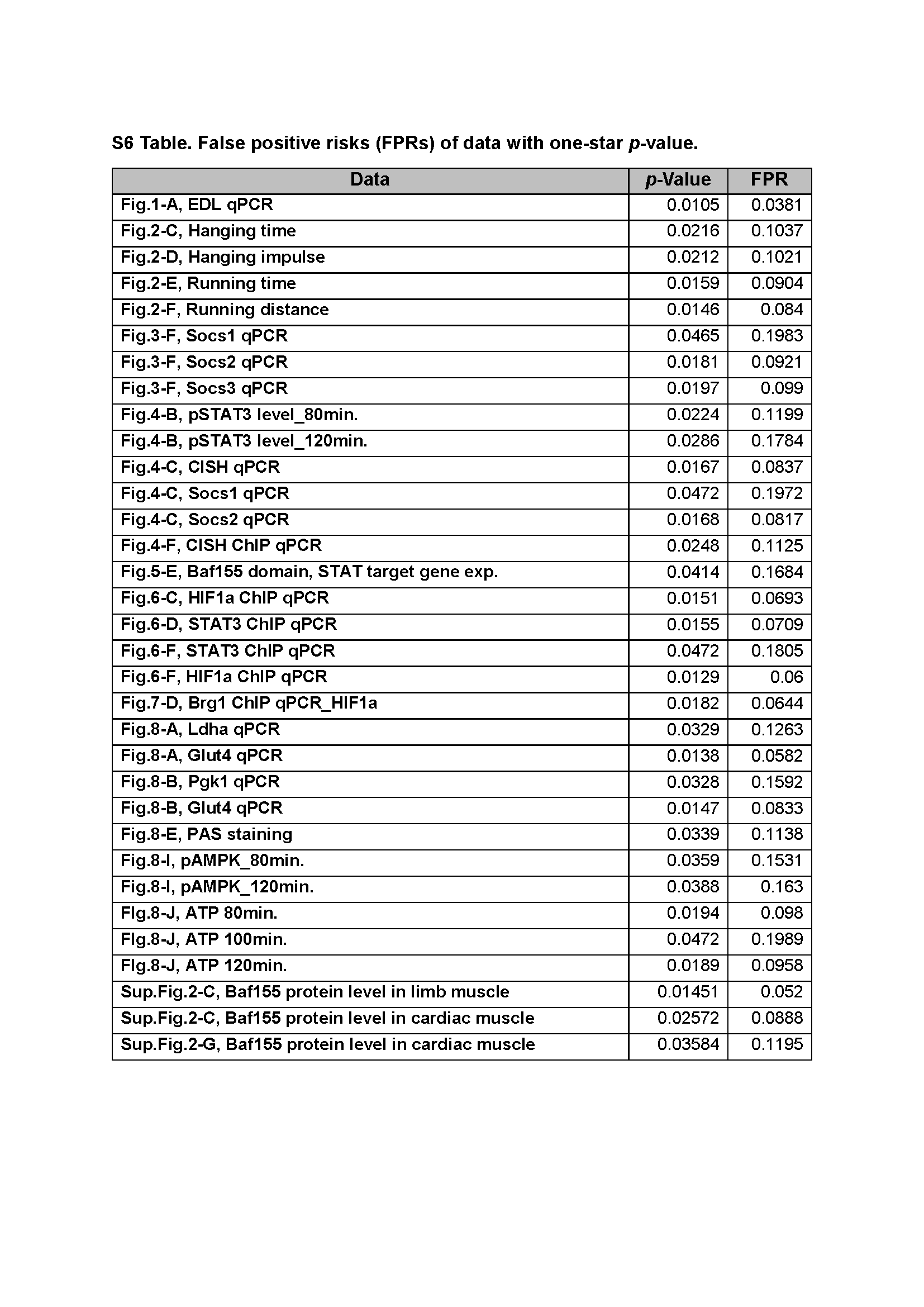

Supplement: S6 Table — The FPRs of the data with one-star p-value (0.01<p<0.05). Information of data was presented as the number of figure-panel label and the purpose of the experiment. (TIF) [file pbio.3002192.s011.tif]

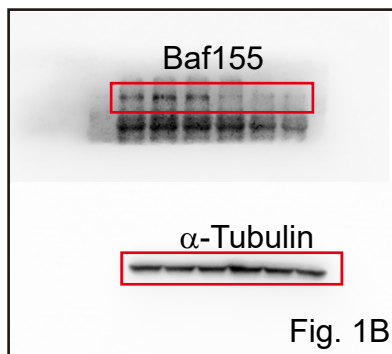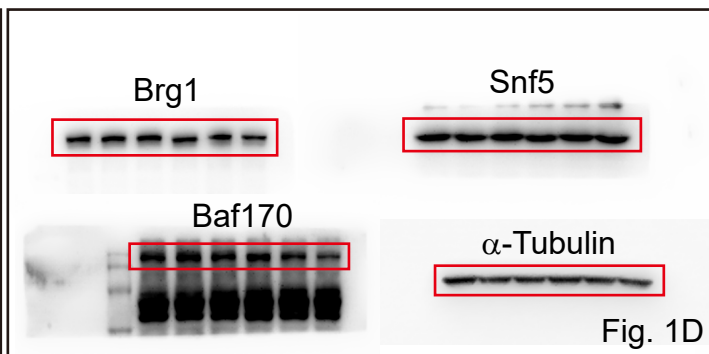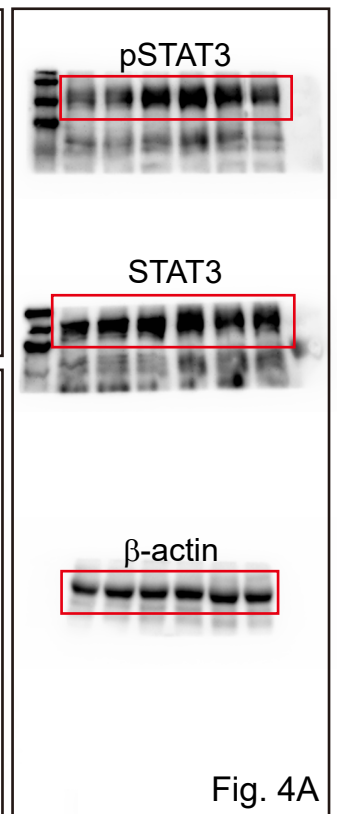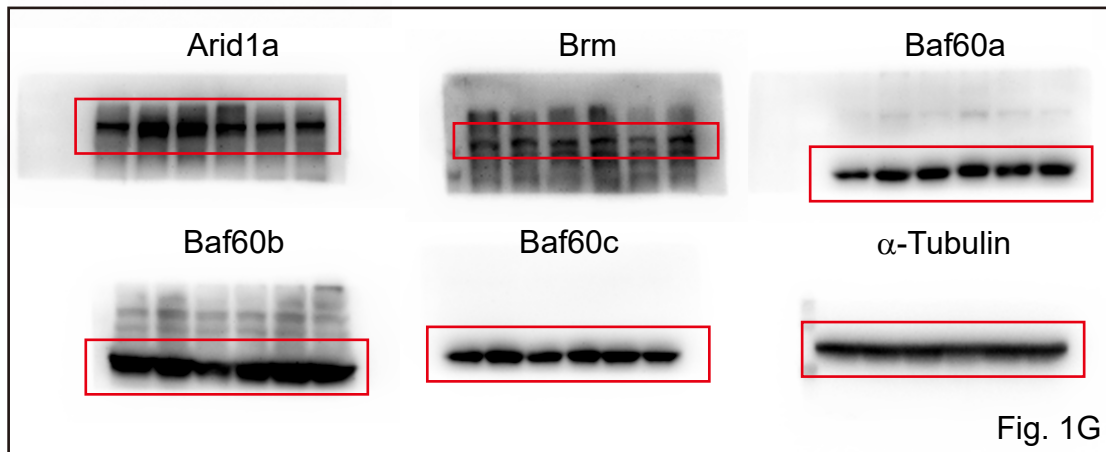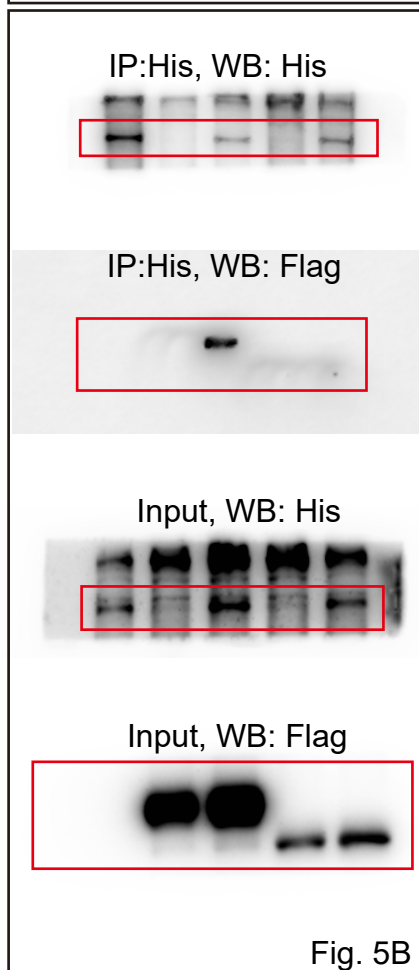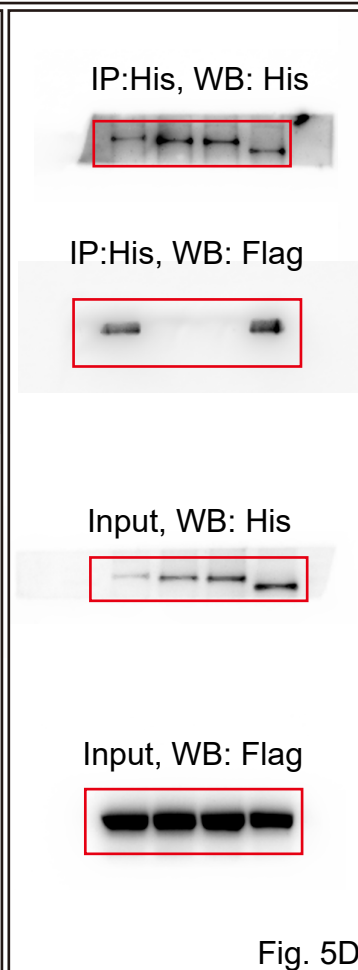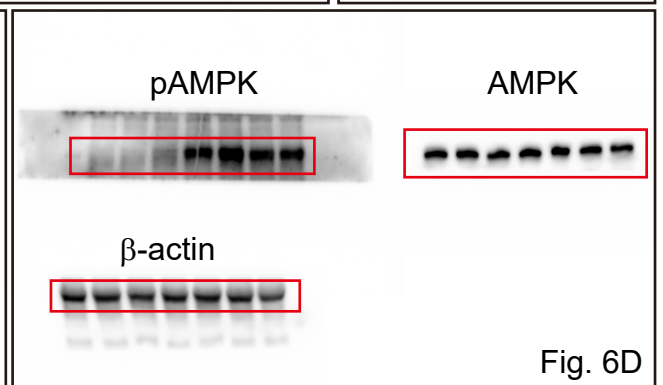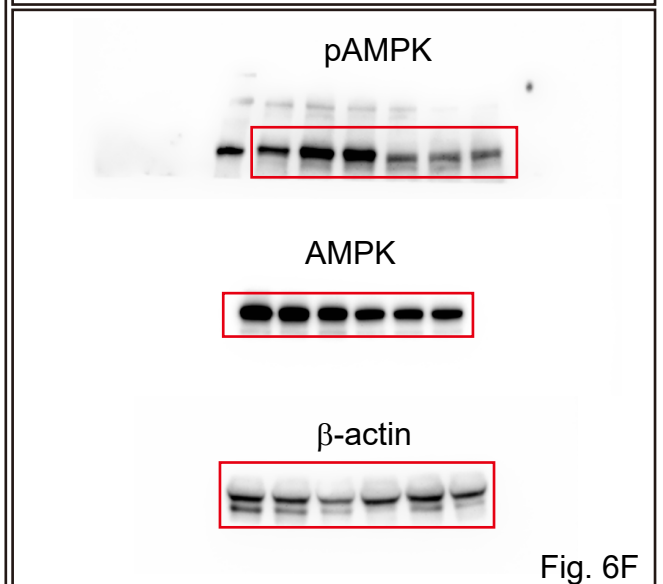

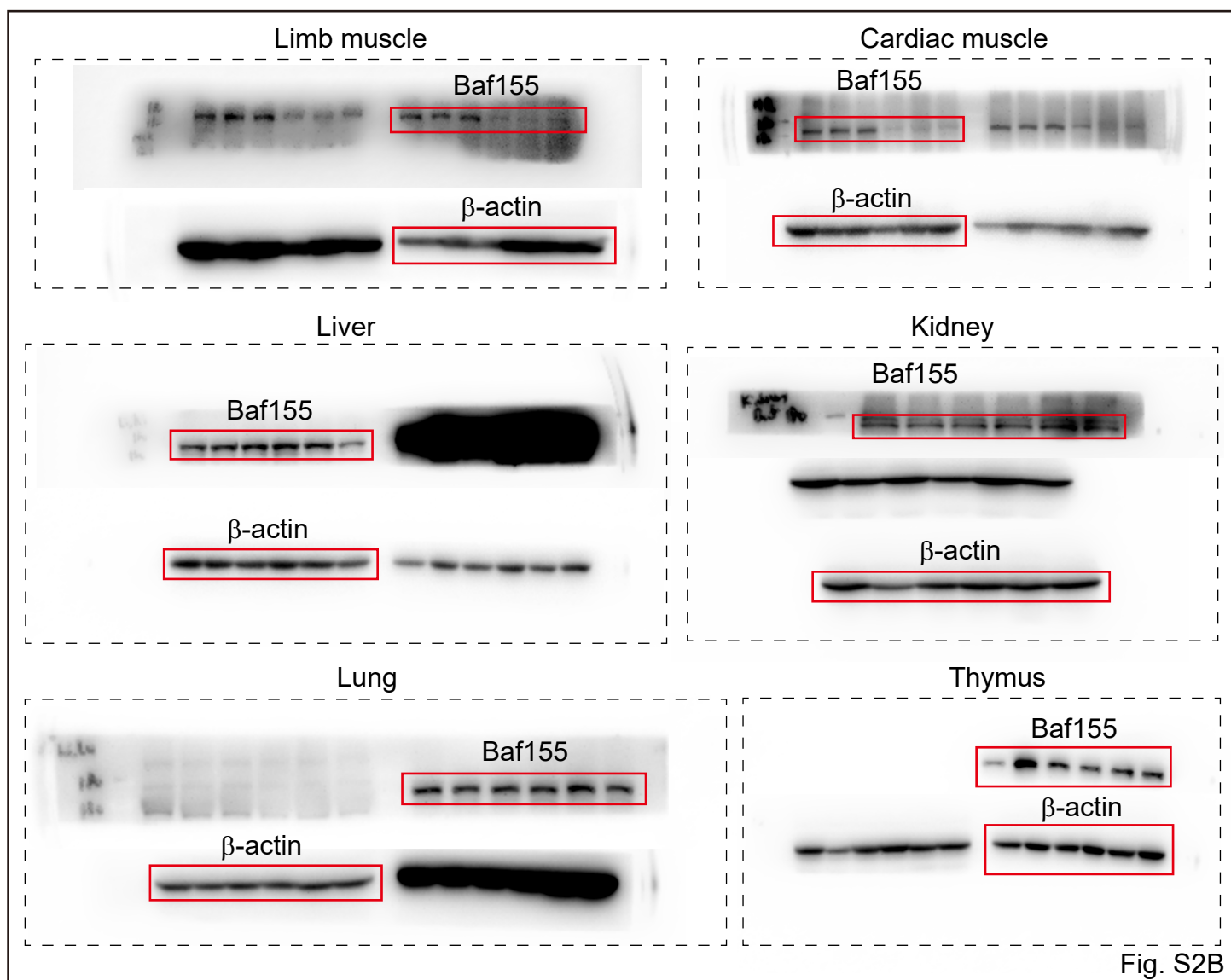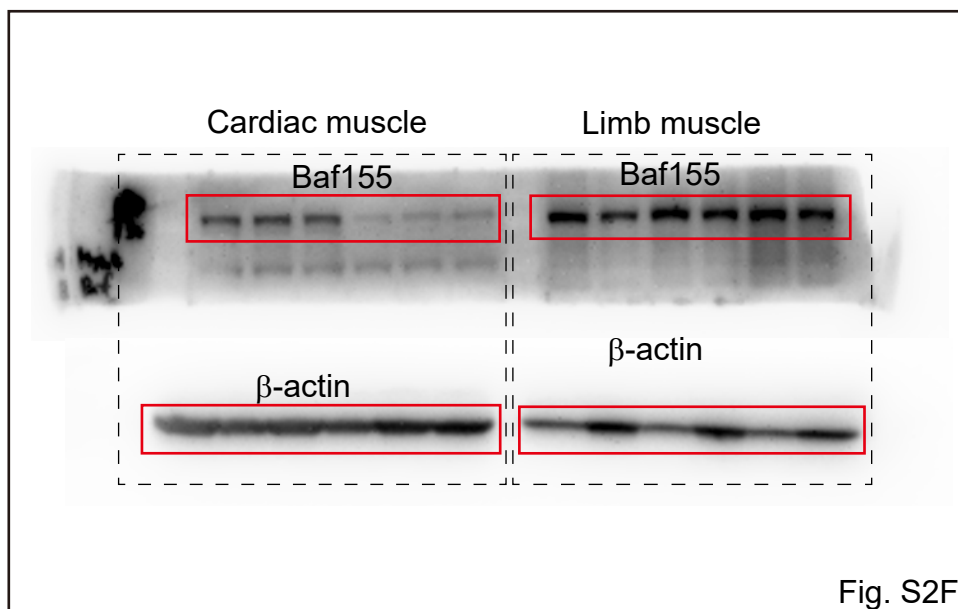

Supplement: S1 Raw Image — The red square within each image indicates the cropped area for the representative image. (PDF) [file pbio.3002192.s013.pdf]
